# Trigonometric Bundling Disulfide Unit Starship Synergizes More Effectively to Promote Cellular Uptake

**DOI:** 10.3390/ijms25147518

**Published:** 2024-07-09

**Authors:** Lei Wang, Dezhi Wang, Wenzhuo Lei, Tiantian Sun, Bei Gu, Han Dong, Yosuke Taniguchi, Yichang Liu, Yong Ling

**Affiliations:** 1 School of Pharmacy, Jiangsu Province Key Laboratory for Inflammation and Molecular Drug Target, Nantong University, Nantong 226001, China; 18962706185@163.com (D.W.); futuredistance2022@163.com (W.L.); stt0715ntu@163.com (T.S.); 13145020630@163.com (B.G.); 13775710235@163.com (H.D.); yichangliu@ntu.edu.cn (Y.L.); 2 School of Pharmaceutical Sciences, Kyushu University, 3-1-1 Maidashi, Higashi-ku, Fukuoka 812-8582, Japan; taniguch@phar.kyushu-u.ac.jp

**Keywords:** disulfide unit, cellular uptake, cytosolic delivery

## Abstract

A small molecule disulfide unit technology platform based on dynamic thiol exchange chemistry at the cell membrane has the potential for drug delivery. However, the alteration of the CSSC dihedral angle of the disulfide unit caused by diverse substituents directly affects the effectiveness of this technology platform as well as its own chemical stability. The highly stable open-loop relaxed type disulfide unit plays a limited role in drug delivery due to its low dihedral angle. Here, we have built a novel disulfide unit starship based on the 3,4,5-trihydroxyphenyl skeleton through trigonometric bundling. The intracellular delivery results showed that the trigonometric bundling of the disulfide unit starship effectively promoted cellular uptake without any toxicity, which is far more than 100 times more active than that of equipment with a single disulfide unit in particular. Then, the significant reduction in cell uptake capacity (73–93%) using thiol erasers proves that the trigonometric bundling of the disulfide starship is an endocytosis-independent internalization mechanism via a dynamic covalent disulfide exchange mediated by thiols on the cell surface. Furthermore, analysis of the molecular dynamics simulations demonstrated that trigonometric bundling of the disulfide starship can significantly change the membrane curvature while pushing lipid molecules in multiple directions, resulting in a significant distortion in the membrane structure and excellent membrane permeation performance. In conclusion, the starship system we built fully compensates for the inefficiency deficiencies induced by poor dihedral angles.

## 1. Introduction

Efficient and safe cellular delivery strategies currently remain one of the great challenges in chemical biology [1]. In particular, the delivery of poorly soluble, highly toxic small molecular entities or natural products and, moreover, large substrates such as nucleic acids and peptide drugs by nanoparticles usually occurs through endocytosis [2,3,4,5,6,7,8,9,10,11,12,13,14,15,16]. However, cellular delivery mediated by endocytosis can easily lead to drug retention by the endosomes, which makes release into the cytoplasm more difficult [17]. In addition, these delivery systems face serious challenges such as high dosage [18] difficulty in unifying the size of vector–drug complexes [19] and high cytotoxicity. Therefore, endocytosis-independent cellular uptake has attracted increasing attention because of its ability to rapidly distribute drugs in the cytoplasm [20]. Currently, dynamic covalent disulfide exchange chemistry at the cell surface is emerging as an innovative strategy to address the above-mentioned challenges [21]. Briefly, a drug coupled with the modification of a disulfide unit undergoes disulfide exchange with thiols on the cell surface, causing the drug to covalently adsorbs on the cell surface and be taken up by the cell. Glutathione reduction in the cytoplasm completes the release of the drug in the cytoplasm. Due to the advantages of low molecular weight, lack of charge and non-toxicity, the active disulfide units have been widely used in the field of drug delivery [22,23,24,25,26,27].

**Figure 1:**
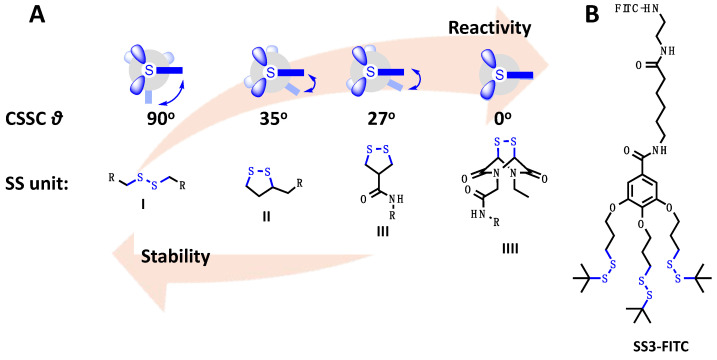
(**A**) Brief description of disulfide unit performance between structure and corresponding dihedral angle. (**B**) The trigonometric bundling disulfide unit starship strategy used.

Dynamic covalent disulfide exchange on the cell surface is a crucial step in this strategy, which directly impacts drug binding and cellular uptake. Therefore, disulfide modification strategies that can effectively promote the efficiency of the covalent exchange of thiols are the focus of current development. First, Matile’s groups [28,29,30] discovered a strong correlation between the CSSC dihedral angle (*θ*) of the disulfide unit and cellular uptake. All reported disulfide units can be divided into three types (Figure 1A): open-loop relaxed type (**I**, *θ* = 90°), ring-comforting type (**II, III** with *θ* of 35° and 27°, respectively), and ring-tensing type (**IIII**, *θ* = 0°). Although there are slight differences in activity between different structures, smaller CSSC dihedral angles generally result in faster disulfide exchange reactions, leading to increased drug attachment to cell surfaces and improved cellular uptake within a shorter time frame [31]. This is also proven by the energy profile analysis (Figure S18) of different disulfide units with different CSSC dihedral angles. However, the reduction in the dihedral angle comes with the risk of polymerization of the disulfide unit [32,33,34,35,36]. Particular care is required in peptide and nucleic acid drug synthesis processes, which often involve strong acidic, alkaline, or oxidation-reduction environments to prevent inactivation caused by polymerization. Therefore, a moderate change in the dihedral angle serves as a foundation for ensuring the stability and intracellular uptake efficiency of coupling complexes. Second, the number and tandem mode between disulfide units and drugs also directly affect the cellular uptake efficiency [37]. Coupling numerous disulfide units indeed enhances intracellular uptake, but a simple and effective tandem approach needs to be further explored.

To further enhance the wide application of open-loop relaxed-type disulfide units in drug delivery, we constructed a trigonometric bundling starship based on a 3,4,5-trihydroxyphenyl skeleton with the reported simple small *tert*-butyl disulfide unit, in which the three contained disulfide units were arranged in the same space at a unique angle (Figure 1B). It is expected to synergistically promote intracellular uptake to compensate for the low exchange rate.

## 2. Results and Discussion

### 2.1. Chemistry

**Scheme 1:**
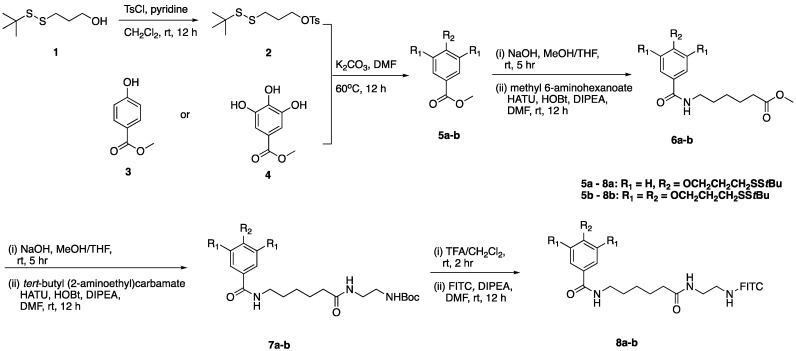
Synthesis of **SS1/3-FITC** probe (**8a** and **8b**).

The synthesis scheme for **SS1/3-FITC** probes is illustrated in Scheme 1. Specifically, the TsCl reagent was utilized to activate the terminal hydroxyl group of the disulfide unit in the presence of pyridine in CH_2_Cl_2_. The mono- or tri-substituted conjugation products **5a-b** were then obtained in high yield using the activated intermediate **2** with mono- or trihydroxybenzoate (**3** or **4**, respectively) under the K_2_CO_3_ condition. After the hydrolysis of methyl benzoate, the linker was extended by classical amide condensation reaction and finally conjugated with FITC under alkaline conditions to obtain fluorescent probes **8a-b** (**SS1-FITC** and **SS3-FITC**, respectively). Finally, the UV absorbance value of FITC at 520 nm in sodium tetraborate buffer (pH = 9.18) was used to calculate the exact concentration of the fluorescent probe. ^1^H NMR, ^13^C NMR, and HRMS spectra of all compounds are provided in Figures S1–S12.

### 2.2. Biological Activity

#### 2.2.1. CCK-8 Assay

**Figure 2:**
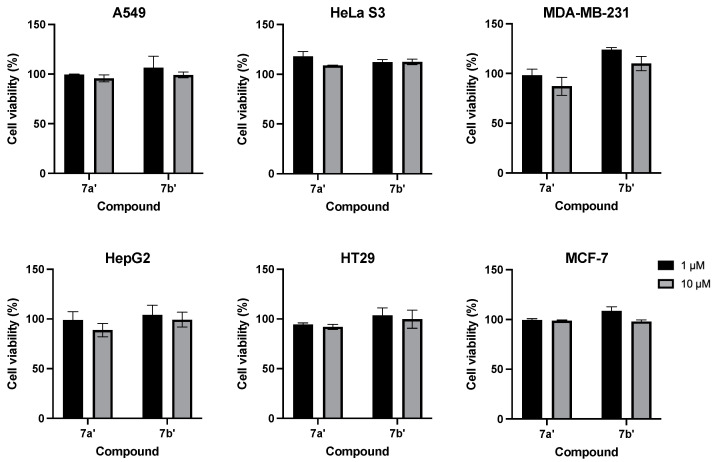
The cytotoxicity of disulfide unit starship using CCK-8 assay for intermediate **7a’** and **7b’**.

Firstly, the CCK-8 assay was used to assess the cytotoxicity of the disulfide unit starship and their intracellular metabolites in cells. To eliminate interference from fluorophores, the intermediate **7a’** and **7b’** (Scheme 1) of the starship were used for specific exercises. The results showed that both their intracellular glutathione hydrolysis products were not cytotoxic in the above commonly used cancer cells, even at 10 μM (Figure 2).

#### 2.2.2. Time-Dependent Cellular Uptake of SS1/3-FITC Probe

**Figure 3:**
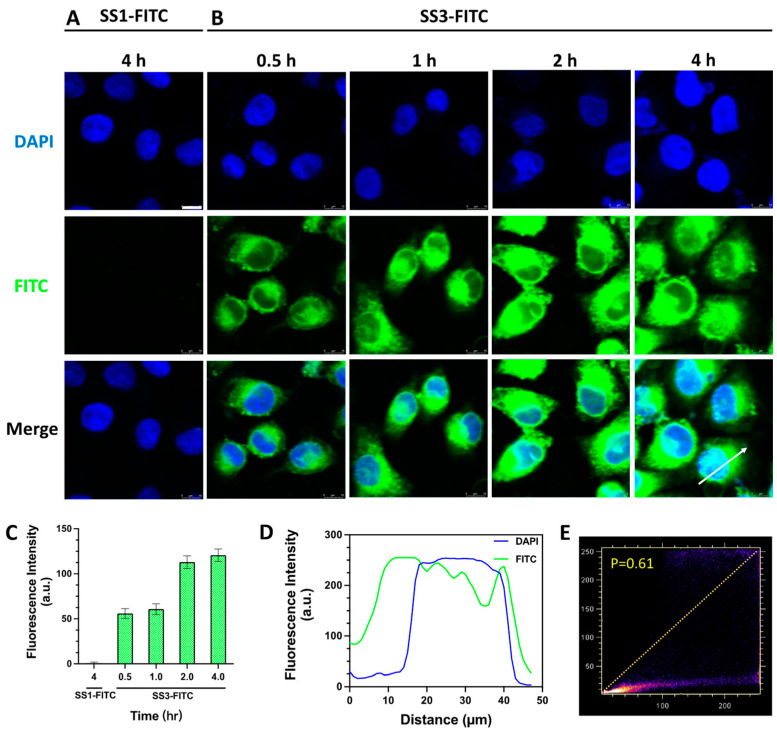
CLSM images of A549 cells after incubation with 1 μM SS1/SS3-FITC probe together with DAPI to stain the nuclei for (**A**,**B**); Quantitative analysis of fluorescence intensity and colocalization analysis are shown in (**C**,**D**), respectively. Scatterplot analysis of the merge image in the last view with the white arrow symbol is shown in (**E**). Scar bar: 10 µm.

A series of cell experiments were performed to evaluate the bioactivity of the fluorescent probes. Firstly, the uptake of the fluorescent probes **SS1-FITC** and **SS3-FITC** into various cancer cells was monitored using confocal laser scanning microscopy (CLSM). The incubation of A549 cells with a 1 μM **SS3-FITC** probe in DMEM medium for 4 h at 37 °C resulted in intense and uniform fluorescence emission from the cytoplasm of each cell (Figure 3B), including poorly stained nuclei with DAPI. At the same time, slightly weak fluorescence emission was also detected in the nuclei of some cells with a *p* value of 0.61 (Figure 3D,E). In sharp contrast, the **SS1-FITC** probe (1 μM) equipped with a single disulfide unit failed to detect significant fluorescence emission after 4 h of incubation with A549 cells (Figure 3A), which was consistent with the low cellular uptake of open-loop relaxed type disulfide units reported previously [37]. To exclude the fluorescence interference of the FITC fluorophore itself, no obvious fluorescence signal was found for the disulfide unit in which the FITC-NH_2_ probe was absent under the same conditions (Figure S13). These results indicate that the trigonometric bundling disulfide unit can effectively promote intracellular uptake. Meanwhile, similar results were observed in several other cell lines (HeLa S3, MDA-MB-231, HepG2, HT29 and MCF-7, Figures S14A,E, S15 and S16, respectively). In total, we used six different tumor cell lines to evaluate the cell uptake effect of fluorescent probes. The results from CLSM showed that SS3-FITC probes exhibited strong cell delivery effects on various cell lines, indicating their potential in the field of anti-tumor drug delivery in the future. Among these cell lines, fluorescence intensity analysis revealed that the SS3-FITC probe demonstrated the highest cellular uptake ability in A549 cells (Figure S17). We hypothesize that differences in the number of thiol groups on the cell membrane or variations in expression levels of thiol-rich proteins may contribute to this discrepancy in cellular uptake efficiency.

We also investigated in detail the uptake status of the **SS3-FITC** probe (1 μM) in A549 cells at different incubation time points (0.5, 1.0, 2.0, 4.0 h) (Figure 3B). It was evident that the **SS3-FITC** probe rapidly distributed in the cell membrane and cytoplasm within 0.5 h incubation, and the intracellular probe concentration increased significantly with the prolongation of the incubation time (Figure 3C). It was found that the fluorescent signal runs in points around the cell membrane. Once inside the cell, the probe was evenly distributed throughout the cytoplasm and part of the nucleus (Figure 3D,E), which is consistent with the previously described mechanism of disulfide unit intracellular transport [31]. In addition, similar results were observed in the HeLa S3 cell line (Figure S14B–E). These results indicate that the designed trigonometric bundling disulfide unit starship not only greatly promotes cellular uptake but also achieves ultrafast cytoplasmic distribution, which has strong potential for clinical application.

#### 2.2.3. Concentration-Dependent Cellular Uptake of SS1/3-FITC Probe

**Figure 4:**
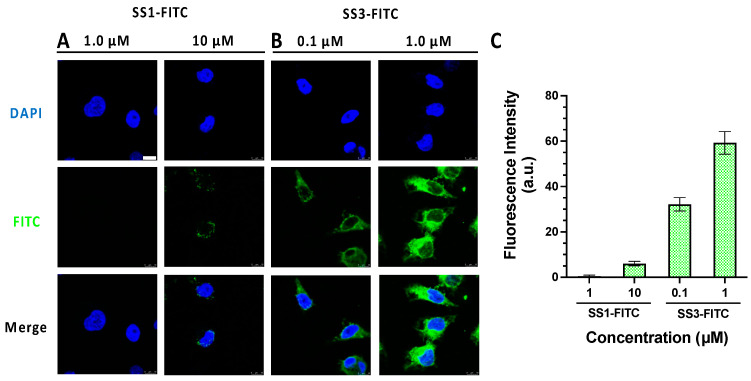
CLSM images of A549 cells after 1 h incubation with different concentrations of **SS1/SS3-FITC** probe together with DAPI to stain the nuclei for (**A**,**B**). Quantitative analysis of fluorescence intensity is shown in (**C**). Scar bar: 10 µm.

To further visualize the comparison of intracellular uptake of the **SS3-FITC** and **SS1-FITC** probes, different concentrations of the fluorescent probes were incubated in A549 cells for 1 h and analyzed by CLSM (Figure 4). Apparently, **SS3-FITC** (0.1 μM) still efficiently entered the cells even at lower concentrations (Figure 4B). However, **SS1-FITC** (10 μM) still had no positive influence on intracellular delivery at high concentration conditions (Figure 4A). The fluorescence intensity obtained at 0.1 μM using the trigonometric bundling disulfide unit starship is still higher than that obtained at 10 μM using the single disulfide unit-equipped transporter, indicating that the trigonometric bundling disulfide unit starship is much more than 100-fold more active (Figure 4C), which was also found in Figure 3C. The nucleic acid drug constructed by the linear disulfide bond coupling method adopted by Abe’s group is upgraded from 1 disulfide unit in tandem to 5 disulfide units in series and then to 10 disulfide units in series, and its activity is about 2–4 times higher [37]. This fully proves the synergistic highly efficient intracellular delivery potential of the trigonometric bundling type disulfide unit starship, which fully compensates for the low reaction speed caused by the dihedral angle.

#### 2.2.4. Cellular Uptake of SS3-FITC Probe with Thiols Inhibitors

**Figure 5:**
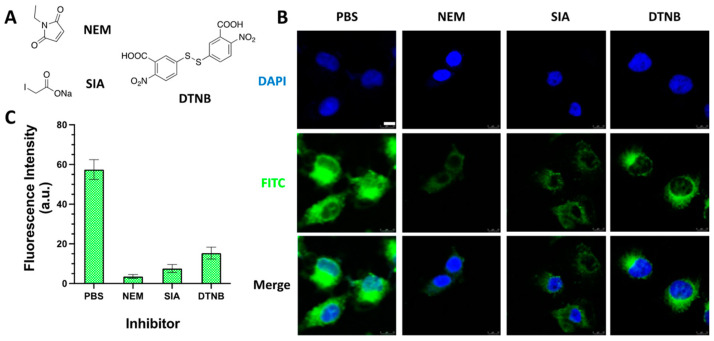
CLSM images of A549 cells that were preincubated with inhibitors (NEM, SIA, and DTNB, 1.2 mM, 0.5 h) and then incubated with an **SS3-FITC** probe (1 μM, 1 h) together with DAPI to stain the nuclei for (**A**,**B**). Quantitative analysis of fluorescence intensity is shown in (**C**). Scar bar: 10 µm.

To further elucidate the cellular uptake mechanism of the trigonometric bundling **SS3-FITC** probe, the following experiments were performed. Previous studies showed that the disulfide unit on the probe first undergoes a chemical exchange reaction with the thiol group of the proteins on the cell membrane. Therefore, the number and status of thiol groups of the proteins on the cell membrane play a key role in the intracellular delivery of the probe. In this study, cells were pretreated with several inhibitors for 0.5 h to reduce the amount and/or state of thiol groups on the cell surface, and intracellular uptake of the probe was investigated (Figure 5A–C). Firstly, the inhibitors *N*-Ethylmaleimide (NEM) [38] and sodium iodoacetate (SIA) [29] were used to pre-treat the thiol groups on the surface of A549 cells, and the uptake capacity of **SS3-FITC** was decreased by 93% and 86%, respectively. Secondly, the uptake capacity of **SS3-FITC** was reduced by 73% by using DTNB to pre-treat A549 cells to convert thiol groups on the cell surface to a disulfide state. These results confirmed that **SS3-FITC** starship is an endocytosis-independent internalization mechanism via a dynamic covalent disulfide exchange mediated by thiols on cell surface proteins.

#### 2.2.5. Molecular Dynamics Simulations Analysis

**Figure 6:**
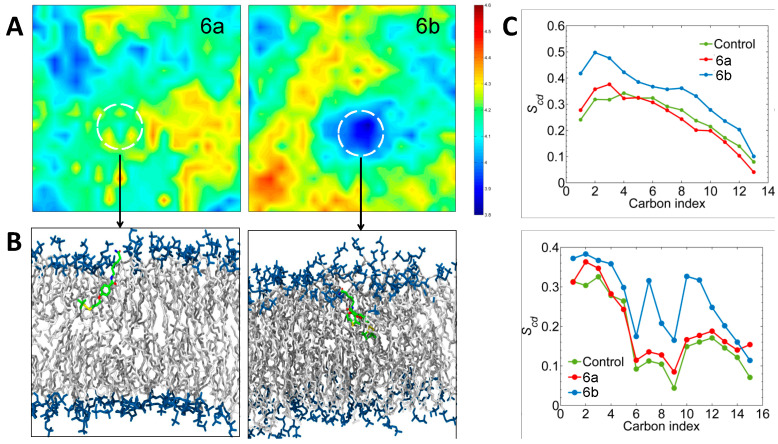
(**A**) The membrane distortion induced by **6a** and **6b**. The local membrane thickness maps were shown in the top panel (color bar unit: nm). (**B**) The molecular details sounded by **6a** and **6b** were shown in the bottom panel (the head groups and tail groups of lipid molecules were colored in sea blue and white; the carbon, oxygen, nitrogen, and sulfur atoms in **6a** and **6b** were colored in green, red, blue, and yellow, respectively). (**C**) The lipid order parameters of (*S*_cd_) of the palmitic chain (up) and oleic chain (down) in lipid molecules. The carbon index started from the carboxyl group of the fatty acid chain. The legend “control” indicates that nothing inserts in membrane.

Having recognized the potential impact of the trigonometric bundling disulfide unit starship on improving cellular uptake, we further examined the deformation of lipid bilayers induced by key intermediates **6a** and **6b** (Scheme 1), which did not present interference from fluorophores. Based on the 500 ns long classical molecular dynamics (MD) simulation trajectories, it was evident that **6a** and **6b** exhibited different cell membrane distortion behaviors. The local membrane thickness maps revealed that **6a** slightly impacted the membrane thickness, while **6b** could significantly change the membrane curvature (Figure 6A). In addition, our simulations demonstrated that both **6a** and **6b** were inserted into the cell membrane via disulfide units. From the viewpoint of structure, the linear structure of **6a** is similar to that of lipid molecules. The insertion of **6a** would not drastically impact the alignment of the lipid molecules. Meanwhile, **6b** could simultaneously squeeze lipid molecules in multiple directions, resulting in significant distortion of the membrane structure (Figure 6B). Moreover, the collapse of the lipid bilayer allowed **6b** to penetrate deeper into the membrane. The orientation of the fatty acid chains of lipids around the small molecules can be monitored using the lipid deuterium order parameter (*S*_cd_) [39]. The *S*_cd_ value of 0 indicates a completely random alignment, while a value of 1 indicates alignment perpendicular to the membrane plane [40]. As shown in Figure 6C, two lipid tails tended to have more random orientations in the presence of **6b**, meaning that **6b** had a stronger disruption effects on the lipid bilayer. Note that disrupting the steady state of the lipid bilayer is a prerequisite for small molecule transport through the membrane; we believe that **6b** had better membrane permeation performance than **6a** [41].

## 3. Materials and Methods

### 3.1. General Method

All chemicals were purchased from commercial suppliers. The ^1^H and ^13^C NMR spectra of synthesized compounds were characterized on a 400 MHz spectrometer (Bruker, Billerica, MA, USA), and high-resolution mass spectra (HRMS) were recorded on a mass spectrometer (Waters, Milford, MA, USA). The fluorescence images of cultured cancer cells were captured by confocal laser scanning microscope (CLSM, Leica, Wetzlar, Germany, TCS SP8) and analyzed by ImageJ (https://imagej.net/software/imagej/ (accessed on 5 July 2024)). The CCK-8 assay was performed on a multifunctional microplate reader (Synergy H1, Bio-Tek, Winooski, VT, USA).

### 3.2. General Method for Preparation of Probe SS1/3-FITC 

#### 3.2.1. Synthesis of 3-(Tert-butyldisulfaneyl)propyl-4-methylbenzenesulfonate (**2**)

To a dried flask was added 3-(tert-butyldisulfaneyl)propan-1-ol (**1**, 1.6 g, 8.87 mmol) in dry dichloromethane (17.7 mL) under an N_2_ atmosphere. The dried pyridine (1.08 mL, 13.31 mmol) and 4-toluenesulfonyl chloride (2.03 g, 10.65 mmol) were successively added to the solvent under the ice bath. The reaction mixture was stirred at room temperature overnight. After the addition of water (20 mL) and a saturated aqueous NH_4_Cl solution (10 mL), the mixture was extracted with CH_2_Cl_2_ (3 × 50 mL). The combined organic extracts were dried over Na_2_SO_4_ and concentrated in vacuo before the purification of the crude product by column chromatography (petroleum ether:ethyl acetate = 60:10), which yielded **2** as a colorless oil (2.2 g, 6.60 mmol, 74%). ^1^H NMR (400 MHz, CDCl_3_) *δ* 7.79 (d, *J* = 8.0 Hz, 2H), 7.36 (d, *J* = 8.0 Hz, 2H), 4.13 (t, *J* = 6.0 Hz, 2H), 2.68 (t, *J* = 7.0 Hz, 2H), 2.46 (s, 3H), 2.02 (p, *J* = 6.5 Hz, 2H), 1.30 (s, 9H). ^13^C NMR (101 MHz, CDCl_3_) *δ* 144.96, 132.73, 129.97, 127.94, 68.63, 48.03, 35.64, 29.92, 28.30, 21.74. ESI-HRMS (*m*/*z*): Calcd. For C_14_H_23_O_3_S_3_ [M+H]^+^: 335.0804, Found: 335.0801.

#### 3.2.2. Synthesis of Ethyl 4-(3-(Tert-butyldisulfaneyl)propoxy)benzoate (**5a**)

Compound **2** (1.86 g, 5.55 mmol) was added to a solution of ethyl 4-hydroxybenzoate (**3**, 1.37 g, 8.25 mmol) and K_2_CO_3_ (1.14 g, 8.25 mmol) in dry DMF (20 mL). The resulting mixture was stirred at 60 °C overnight under an N_2_ atmosphere. Next, ethyl acetate (200 mL) was added, and the organic phase was washed with brine (3 × 30 mL) and dried over Na_2_SO_4_ before the solvent was removed under reduced pressure. Purification by column chromatography (petroleum ether:ethyl acetate = 40:1) yielded the title compound as a pale-yellow oil (**5a**, 1.05 g, 3.20 mmol, 58%). ^1^H NMR (400 MHz, CDCl_3_) *δ* 7.98 (d, *J* = 8.8 Hz, 2H), 6.90 (d, *J* = 8.8 Hz, 2H), 4.34 (q, *J* = 7.1 Hz, 2H), 4.11 (t, *J* = 6.0 Hz, 2H), 2.87 (t, *J* = 7.0 Hz, 2H), 2.18 (p, *J* = 6.5 Hz, 2H), 1.38 (t, *J* = 7.1 Hz, 3H), 1.38 (s, 9H). ^13^C NMR (101 MHz, CDCl_3_) *δ* 166.44, 162.54, 131.55, 122.94, 114.04, 66.07, 60.67, 47.99, 36.62, 29.97, 28.66, 14.41. ESI-HRMS (*m*/*z*): Calcd. For C_16_H_25_O_3_S_2_ [M+H]^+^: 329.1240, Found: 329.1238.

#### 3.2.3. Synthesis of Methyl 3,4,5-tris(3-(Tert-butyldisulfaneyl)propoxy)benzoate (**5b**)

Compound **2** (2.2 g, 6.60 mmol) was added to a solution of methyl 3,4,5-trihydroxybenzoate (**4**, 0.37 g, 2.00 mmol) and K_2_CO_3_ (1.11 g, 4.00 mmol) in dry DMF (10 mL). The resulting mixture was stirred at 60 °C overnight under an N_2_ atmosphere. Next, ethyl acetate (200 mL) was added, and the organic phase was washed with brine (3 × 30 mL) and dried over Na_2_SO_4_ before the solvent was removed under reduced pressure. Purification by column chromatography (petroleum ether:ethyl acetate = 20:1) yielded the title compound as a pale-yellow oil (**5b**, 0.98 g, 1.46 mmol, 73%). ^1^H NMR (400 MHz, CDCl_3_) *δ* 7.29 (s, 2H), 4.16–4.10 (m, 6H), 3.91 (s, 3H), 2.98–2.89 (m, 6H), 2.26–2.10 (m, 6H), 1.36 (s, 27H). ^13^C NMR (101 MHz, CDCl_3_) *δ* 168.26, 166.73, 154.83, 152.78, 152.43, 143.86, 141.57, 133.61, 125.07, 119.93, 108.71, 107.90, 72.36, 71.82, 71.48, 67.07, 66.88, 52.51, 52.29, 48.04, 47.98, 47.90, 47.86, 47.84, 42.05, 37.17, 36.91, 36.84, 36.77, 36.63, 35.02, 31.03, 30.04, 30.00, 28.79, 27.13. ESI-HRMS (*m*/*z*): Calcd. For C_29_H_51_O_5_S_6_ [M+H]^+^: 671.2055, Found: 671.2053.

#### 3.2.4. Synthesis of Methyl 6-(4-(3-(Tert-butyldisulfaneyl)propoxy)benzamido) Hexanoate (**6a**)

To a solution of compound **5a** (1.00 g, 3.04 mmol) in MeOH/THF (1:1, 15 mL) was added 2N aqueous NaOH (0.61 g, 15.22 mmol). The resulting mixture was stirred at room temperature for 5 h. After adding water (15mL), the pH was neutralized to 4.0 by 1N HCl solution. The mixture was extracted with EtOAc (3 × 50 mL). The combined organic extracts were dried over Na_2_SO_4_ and concentrated in vacuo to yield compound **6a’** as a white solid (0.90 g, 3.00 mmol, 99%) without purification for the next step.

To a dried flask was added compound **6a’** (0.90 g, 3.0 mmol) and DIPEA (1.04 mL, 6.00 mmol) in dry CH_2_Cl_2_ (30 mL) under an N_2_ atmosphere. The HOBt (0.81 g, 6.00 mmol) and HATU (1.37 g, 3.60 mmol) were successively added to the solvent, which was followed by the addition of methyl 6-aminohexanoate hydrochloride (0.82 g, 4.50 mmol) after 30 min. The reaction mixture was stirred at room temperature overnight. After adding water (100 mL), the mixture was extracted with CH_2_Cl_2_ (3 × 30 mL). The combined organic extracts were dried over Na_2_SO_4_ and concentrated in vacuo before the purification of the crude product by column chromatography (petroleum ether:ethyl acetate = 2:1), which yielded compound **6a** as a colorless oil (1.31 g, 3.00 mmol, 100%). ^1^H NMR (400 MHz, CDCl_3_) *δ* 7.71 (d, *J* = 8.7 Hz, 2H), 6.90 (d, *J* = 8.8 Hz, 2H), 6.16 (t, *J* = 5.7 Hz, 1H), 4.10 (m, 2H), 3.66 (s, 3H), 3.43 (q, *J* = 6.7 Hz, 2H), 2.87 (t, *J* = 7.0 Hz, 2H), 2.33 (t, *J* = 7.3 Hz, 2H), 2.20–2.14 (m, 2H), 1.74–1.58 (m, 4H), 1.44–1.39 (m, 2H), 1.33 (s, 9H).

#### 3.2.5. Synthesis of Methyl 6-(3,4,5-tris(3-(Tert-butyldisulfaneyl)propoxy)benzamido) Hexanoate (**6b**)

To a solution of compound **5b** (0.98 g, 1.46 mmol) in MeOH/THF (2:1, 15 mL) was added 2N aqueous NaOH (0.29 g, 7.30 mmol). The resulting mixture was stirred at room temperature for 5 h. After the addition of water (15 mL), the pH was neutralized to 4.0 by 1N HCl solution. The mixture was extracted with EtOAc (3 × 30 mL). The combined organic extracts were dried over Na_2_SO_4_ and concentrated in vacuo to yield compound **6b’** as a white solid (0.97 g, 1.46 mmol, 100%) without purification for the next step. ^1^H NMR (400 MHz, CDCl_3_) *δ* 7.19 (s, 2H), 4.08–4.04 (m, 6H), 2.90–2.81 (m, 6H), 2.18–2.00 (m, 6H), 1.28 (s, 27H). ESI-HRMS (*m*/*z*): Calcd. For C_28_H_48_O_5_S_6_Na [M+Na]^+^: 679.1718, Found: 679.1719.

To a dried flask was added compound **6b’** (0.2 g, 0.30 mmol) and DIPEA (160 μL, 0.91 mmol) in dry DMF (3.0 mL) under an N_2_ atmosphere. The HOBt (82 mg, 0.61 mmol) and HATU (139 mg, 0.37 mmol) were successively added to the solvent, which was followed by the addition of methyl 6-aminohexanoate hydrochloride (83 mg, 0.46 mmol) after 30 min. The reaction mixture was stirred at room temperature overnight. After the addition of water (10 mL), the mixture was extracted with CH_2_Cl_2_ (3 × 10 mL). The combined organic extracts were dried over Na_2_SO_4_ and concentrated in vacuo before the purification of the crude product by column chromatography (petroleum ether:ethyl acetate = 2.5:1), which yielded compound **6b** as a colorless oil (0.2 g, 0.26 mmol, 85%). ^1^H NMR (400 MHz, CDCl_3_) *δ* 7.01 (s, 1H), 4.17–4.07 (m, 6H), 3.68 (s, 2H), 3.46 (q, *J* = 6.7 Hz, 1H), 2.98–2.89 (m, 8H), 2.36 (t, *J* = 6.0 Hz, 2H), 2.22 (p, *J* = 6.0 Hz, 4H), 2.10 (p, *J* = 6.0 Hz, 2H), 1.72–1.68 (m, 2H), 1.46–1.44 (m, 2H), 1.36 (s, 27H). ^13^C NMR (101 MHz, CDCl_3_) *δ* 174.17, 152.68, 140.40, 130.49, 105.64, 105.26, 71.52, 67.21, 53.46, 51.59, 48.01, 47.80, 39.84, 37.01, 36.80, 33.83, 30.58, 30.04, 30.00, 29.88, 29.25, 28.77, 26.38, 24.39. ESI-HRMS (*m*/*z*): Calcd. For C_35_H_62_O_6_S_6_ [M+H]^+^: 784.2896, Found: 784.2897.

#### 3.2.6. Synthesis of Tert-butyl (2-(6-(4-(3-(tert-butyldisulfaneyl)propoxy)benzamido)hexan-amido)ethyl)carbamate (**7a**)

To a solution of compound **6a** (1.3 g, 3.00 mmol) in MeOH/THF (1:1, 30 mL) was added 2N aqueous NaOH (0.6 g, 15 mmol). The resulting mixture was stirred at room temperature for 2 h. After adding water (30 mL), the pH was neutralized to 4.0 by 1N HCl solution. The mixture was extracted with EtOAc (3 × 50 mL). The combined organic extracts were dried over Na_2_SO_4_ and concentrated in vacuo to yield compound **7a’** as a white solid (1.22 g, 2.95 mmol, 98%) without purification for the next step. ^1^H NMR (400 MHz, CDCl_3_) *δ* 7.72 (d, *J* = 8.8 Hz, 2H), 6.90 (d, *J* = 8.7 Hz, 2H), 6.29 (t, *J* = 5.8 Hz, 1H), 4.09 (t, *J* = 6.0 Hz, 2H), 3.48–3.41 (m, 2H), 2.87 (t, *J* = 7.0 Hz, 2H), 2.36 (t, *J* = 7.3 Hz, 2H), 2.17 (p, *J* = 6.5 Hz, 2H), 1.72–1.59 (m, 4H), 1.46–1.40 (m, 2H), 1.33 (s, 9H). ^13^C NMR (101 MHz, CDCl_3_) *δ* 178.32, 167.33, 161.41, 128.71, 126.83, 114.25, 66.05, 47.99, 39.77, 38.67, 36.64, 33.81, 29.97, 29.30, 28.66, 26.33, 24.28. ESI-HRMS (*m*/*z*): Calcd. For C_20_H_32_NO_4_S_2_ [M+H]^+^: 414.1767, Found: 414.1768.

To a dried flask was added compound **7a’** (150 mg, 0.36 mmol) and DIPEA (0.126 mL, 0.72 mmol) in dry DMF (3.60 mL) under an N_2_ atmosphere. The HOBt (74 mg, 0.54 mmol) and HATU (165 mg, 0.44 mmol) were successively added to the solvent, which was followed by the addition of *tert*-butyl (2-aminoethyl)carbamate (116 mg, 0.72 mmol) after 30 min. The reaction mixture was stirred at room temperature overnight. After adding water (30 mL), the mixture was extracted with CH_2_Cl_2_ (3 × 10 mL). The combined organic extracts were dried over Na_2_SO_4_ and concentrated in vacuo before the purification of the crude product by column chromatography (CH_2_Cl_2_:MeOH = 20:1), which yielded compound **7a** as a white solid (200 mg, 0.36 mmol, 100%). ^1^H NMR (400 MHz, CDCl_3_) *δ* 7.69 (d, *J* = 8.3 Hz, 2H), 6.80 (d, *J* = 8.3 Hz, 2H), 6.75 (brs, 2H), 5.30 (brs, 1H), 4.01 (t, *J* = 6.0 Hz, 2H), 3.35–3.14 (m, 6H), 2.80 (t, *J* = 7.0 Hz, 2H), 2.13–2.08 (m, 4H), 1.58–1.49 (m, 4H), 1.34 (s, 9H), 1.26 (s, 9H). ^13^C NMR (101 MHz, CDCl_3_) *δ* 174.10, 167.44, 161.38, 156.96, 128.82, 126.76, 126.66, 114.16, 79.57, 66.03, 47.98, 40.54, 40.32, 39.67, 36.60, 36.24, 29.96, 29.11, 28.63, 28.39, 26.30, 25.00. ESI-HRMS (*m*/*z*): Calcd. For C_27_H_45_N_3_O_5_S_2_ [M+H]^+^: 556.2874, Found: 556.2875.

#### 3.2.7. Synthesis of Tert-butyl (2-(6-(3,4,5-tris(3-(tert-butyldisulfaneyl)propoxy)benzamido)hexan-amido)ethyl)carbamate (**7b**)

To a solution of compound **6b** (0.2 g, 0.26 mmol) in MeOH/THF (2:1, 3 mL) was added 2N aqueous NaOH (51 mg, 1.28 mmol). The resulting mixture was stirred at room temperature for 2 h. After adding water (5 mL), the pH was neutralized to 4.0 by 1N HCl solution. The mixture was extracted with EtOAc (3 × 10 mL). The combined organic extracts were dried over Na_2_SO_4_ and concentrated in vacuo to yield compound **7b’** as a white solid (0.19 g, 0.25 mmol, 97%) without purification for the next step. ^1^H NMR (400 MHz, CDCl_3_) *δ* 7.01 (s, 2H), 4.19–4.07 (m, 6H), 3.48 (q, *J* = 6.7 Hz, 2H), 2.98–2.89 (m, 6H), 2.41 (t, *J* = 6.0 Hz, 2H), 2.21 (q, *J* = 6.7 Hz, 4H), 2.12–2.06 (m, 2H), 1.48–1.44 (m, 4H), 1.36 (s, 27H). ^13^C NMR (100 MHz, CDCl_3_) *δ* 178.14, 167.32, 152.68, 140.41, 131.17, 129.99, 128.90, 105.65, 77.26, 71.52, 67.19, 52.72, 48.02, 47.80, 39.87, 37.00, 36.79, 33.63, 30.58, 30.04, 30.00, 29.88, 29.72, 29.23, 28.75, 26.29, 24.16, 22.72, 14.16. ESI-HRMS (*m*/*z*): Calcd. for C_34_H_61_NO_6_S_6_ [M+H]^+^: 770.2739, Found: 770.2740.

To a dried flask was added compound **7b’** (200 mg, 0.26 mmol) and DIPEA (0.09 mL, 0.52 mmol) in dry DMF (2.6 mL) under an N_2_ atmosphere. The HOBt (53 mg, 0.39 mmol) and HATU (118 mg, 0.31 mmol) were successively added to the solvent, which was followed by the addition of *tert*-butyl (2-aminoethyl)carbamate (83 mg, 0.52 mmol) after 30 min. The reaction mixture was stirred at room temperature overnight. After adding water (30 mL), the mixture was extracted with CH_2_Cl_2_ (3 × 10 mL). The combined organic extracts were dried over Na_2_SO_4_ and concentrated in vacuo before the purification of the crude product by column chromatography (CH_2_Cl_2_:MeOH = 20:1), which yielded compound **7b** as a white foam (200 mg, 0.022 mmol, 84%). ^1^H NMR (400 MHz, CDCl_3_) *δ* 6.96 (s, 2H), 4.06 (t, *J* = 6.1 Hz, 4H), 3.99 (t, *J* = 5.9 Hz, 2H), 3.38 (q, *J* = 6.4 Hz, 2H), 3.27 (q, *J* = 5.5 Hz, 2H), 3.18 (m, 2H), 2.87 (t, *J* = 7.1 Hz, 2H), 2.82 (t, *J* = 7.0 Hz, 4H), 2.17–2.10 (m, 6H), 2.01 (p, *J* = 6.4 Hz, 2H), 1.64–1.55 (m, 6H), 1.36 (s, 9H), 1.27 (s, 27H). ^13^C NMR (101 MHz, CDCl_3_) *δ* 173.69, 167.16, 157.02, 152.60, 140.29, 130.03, 105.77, 79.69, 71.49, 67.16, 53.48, 47.98, 47.78, 40.75, 40.28, 39.77, 36.99, 36.79, 36.26, 31.03, 30.03, 30.00, 29.88, 29.70, 29.03, 28.79, 28.39, 26.31, 24.85. ESI-HRMS (*m*/*z*): Calcd. For C_41_H_74_N_3_O_7_S_6_ [M+H]^+^: 912.3846, Found: 912.3843.

#### 3.2.8. Synthesis of SS1-FITC (**8a**)

The compound **7a** (30 mg, 0.054 mmol) was dissolved in the mixture solution of dry CH_2_Cl_2_ and TFA (1.25 mL, 4:1). The mixture was stirred at room temperature for another 0.5 h. To obtain the Boc-deprotected intermediate, all solvent and residual TFA were removed by evaporation with toluene several times. After drying under vacuum conditions, to the solution of the Boc-deprotected intermediated in dry DMF (1 mL) was added DIPEA (14 μL, 0.081 mmol) and FITC (21mg, 0.054 mmol) under an N_2_ atmosphere. The reaction mixture was stirred at room temperature overnight. After adding water (10 mL), the mixture was extracted with CH_2_Cl_2_ (3 × 10 mL). The combined organic extracts were dried over Na_2_SO_4_ and concentrated in vacuo before the purification of the crude product by column chromatography (CH_2_Cl_2_:MeOH:AcOH = 100:10:1), which yielded **SS1-FITC** (**8a**) as a yellow solid (38 mg, 0.045 mmol, 83%). ^1^H NMR (400 MHz, DMSO-*d_6_*) *δ* 7.95–7.79 (m, 4H), 7.07–6.94 (m, 3H), 6.70–6.34 (m, 6H), 4.10–4.07 (m, 2H), 3.22–3.17 (m, 4H), 2.07–2.01 (m, 4H), 1.76 (m, 6H), 1.51–1.45 (m, 4H), 1.29 (s, 9H). ^13^C NMR (101 MHz, DMSO-*d_6_*) *δ* 171.72, 164.82, 161.73, 159.91, 129.25, 128.67, 128.35, 128.32, 126.29, 121.38, 120.40, 113.23, 111.36, 109.85, 109.35, 101.67, 65.21, 47.10, 38.43, 38.22, 35.32, 35.19, 34.72, 30.68, 30.16, 29.00, 28.40, 27.72, 25.56, 24.33, −0.50. ESI-HRMS (*m*/*z*): Calcd. For C_43_H_49_N_4_O_8_S_3_ [M+H]^+^: 845.2707, Found: 845.2710.

#### 3.2.9. Synthesis of SS3-FITC (**8b**)

The compound **7b** (60 mg, 0.066 mmol) was dissolved in the mixture solution of dry CH_2_Cl_2_ and TFA (1.25 mL, 4:1). The mixture was stirred at room temperature for another 0.5 h. To obtain the Boc-deprotected intermediate, all solvent and residual TFA were removed by evaporation with toluene several times. After drying under vacuum conditions, to the solution of the Boc-deprotected intermediated in dry DMF (1 mL) was added DIPEA (17 μL, 0.099 mmol) and FITC (26 mg, 0.066 mmol) under an N_2_ atmosphere. The reaction mixture was stirred at room temperature overnight. After adding water (10 mL), the mixture was extracted with CH_2_Cl_2_ (3 × 10 mL). The combined organic extracts were dried over Na_2_SO_4_ and concentrated in vacuo before the purification of the crude product by column chromatography (CH_2_Cl_2_:MeOH:AcOH = 100:10:1), which yielded **SS3-FITC** (**8b**) as a yellow solid (37 mg, 0.031 mmol, 46%). ^1^H NMR (400 MHz, DMSO-*d*_6_) *δ* 8.44–8.41 (m, 1H), 8.24 (d, *J* = 2.0 Hz, 1H), 8.03 (m, 1H), 7.77 (m, 1H), 7.18–7.16 (m, 3H), 6.67–6.53 (m, 6H), 4.07 (t, *J* = 6.0 Hz, 4H), 3.97 (t, *J* = 6.0 Hz, 2H), 3.55 (m, 2H), 3.27–3.21 (m, 4H), 2.93–2.88 (m, 6H), 2.12–2.05 (m, 6H), 1.99–1.92 (m, 2H), 1.54–1.44 (m, 4H), 1.30 (s, 27H). ^13^C NMR (101 MHz, DMSO-*d*_6_) *δ* 165.73, 152.29, 139.46, 130.21, 129.71, 106.12, 102.74, 71.32, 67.21, 48.16, 48.06, 38.10, 36.84, 36.73, 35.79, 31.20, 30.12, 30.09, 29.52, 29.13, 26.68, 25.48. ESI-HRMS (*m*/*z*): Calcd. For C_57_H_77_N_4_O_10_S_7_ [M+H]^+^: 1201.3679, Found: 1201.3676.

### 3.3. Cell Culture

The cancer cells (A549, HeLa S3, MDA-MB-231, HepG2, MCF-7, and HT29) were grown in the complete medium (DMEM containing 10% FBS, 100 units/mL of penicillin, and 100 μg/mL of streptomycin, Adamas life, Shanghai, China). Cells were cultured in a 5% CO_2_ humidified incubator at 37 °C. Cells were harvested by treating with 0.1% trypsin (Gibco, Waltham, MA, USA, diluted with PBS) and passaged once in 3–4 days.

### 3.4. Time-Dependent Fluorescence Microscopy Measurement of the Cancer Cells Treated with the SS1-FITC and SS3-FITC Probe

The cancer cells (A549, HeLa S3, MDA-MB-231, HepG2, HT29, and MCF-7, 1 × 10^4^ cells) were seeded onto 35 mm glass bottom culture dishes (Biosharp, Guangzhou, China) and grown in the complete medium (DMEM, high glucose, 10% FBS, 1% antibiotics) 24 h before probe treatment. The cells were treated with **SS1-FITC** (1 µM) and **SS3-FITC** (1 µM) probes, respectively, in DMEM at 37 °C for 0.5, 1.0, 2.0, and 4.0 h. After that, the cells were washed with PBS (1 mL × 3) and fixed with 4% PFA for 15 min. All cells were stained with DAPI for 15 min followed by PBS wash (1 mL × 3). The cells were immediately analyzed using a fluorescence microscope (Leica TCS SP8). 

### 3.5. Concentration-Dependent Fluorescence Microscopy Measurement of the Cancer Cells Treated with the SS1-FITC and SS3-FITC Probe

The A549 cells (1 × 10^4^ cells) were seeded onto 35 mm glass-bottom culture dishes (Biosharp) and grown in the complete medium (DMEM, high glucose, 10% FBS, 1% antibiotics) 24 h before probe treatment. The cells were treated with **SS1-FITC** (1 and 10 µM) and **SS3-FITC** (0.1 and 1 µM) probes, respectively, in DMEM at 37 °C for 1.0 h. After that, the cells were washed with PBS (1 mL × 3) and fixed with 4% PFA for 15 min. All cells were stained with DAPI for 15 min followed by PBS wash (1 mL × 3). The cells were immediately analyzed using a fluorescence microscope (Leica TCS SP8).

### 3.6. CCK-8 Assay

The cancer cells (A549, HeLa S3, MDA-MB-231, HepG2, HT29, and MCF-7, 3000–5000 cells/well) were seeded onto a 96-well plate (Labselect) and grown in the complete medium (DMEM, high glucose, 10% FBS, 1% antibiotics, 100 µL) 24 h before probe treatment. The cells were treated with **7a’** (1 and 10 µM, 100 µL) and 7**b’** (1 and 10 µM, 100 µL), respectively, in DMEM at 37 °C for 72 h. The medium was removed, and 20 μL of CCK-8 solution (Adamas life) was added to each plate; the cells were then incubated at 37 °C for 2 h. A multifunctional microplate reader (Synergy H1, Bio-Tek, USA) was used to record the absorbance of the solution in each well at 450 nm.

### 3.7. Fluorescence Microscopy Measurement of the Cancer Cells Pretreated with Thiols Inhibitors

The A549 cells (1 × 10^4^ cells) were seeded onto 35 mm glass-bottom culture dishes (Biosharp) and grown in the complete medium (DMEM, high glucose, 10% FBS, 1% antibiotics) 24 h before probe treatment. The cells were pretreated with **N-ethylmaleimide** (1.2 mM), **sodium iodoacetate** (1.2 mM), and **DTNB** (1.2 mM), respectively, in DMEM at 37 °C for 0.5 h. After that, the cells were then treated with **SS3-FITC** (1 µM) probes in DMEM at 37 °C for 1 h. The cells were washed with PBS (1 mL × 3) and fixed with 4% PFA for 15 min. All cells were stained with DAPI for 15 min followed by PBS wash (1 mL × 3). The cells were immediately analyzed using a fluorescence microscope (Leica TCS SP8).

### 3.8. Molecular Dynamics Simulations

The force field parameters (Lennard–Jones parameters and charge parameters) of **6a** and **6b** were generated using the Antechamber protocol. The lipid bilayer model, composed of 200 palmitoyl-oleoyl-phosphocholine (POPC) molecules (100 per leaflet), was constructed using the CHARMM-GUI (https://charmm-gui.org/ (accessed on 5 July 2024)) Membrane Builder. The TIP3P water model was added to hydrate the lipid bilayer, and all MD systems were neutralized by a concentration of 0.15 M NaCl. For each simulation box, **6a** or **6b** was initially immersed in the bulk solution and 1.5 nm higher than the membrane plane.

All-atom MD simulations were performed in the NP*γ*T ensemble using the AMBER18 software with the GPU acceleration. The Lipid14 force field and Generalized Amber Force Field (GAFF) were used for the POPC and **6a**/**6b**, respectively. A cutoff value of 10 Å was used to treated the nonbonded interactions. The Particle Mesh Ewald (PME) algorithm was used to compute long-range electrostatic interactions. Pressure coupling was maintained at 1 bar using semi-isotropic coupling with the Berendsen barostat. The temperature was controlled by the Nose–Hoover algorithm. The constant surface tension is used in statistical ensembles for the simulations of the aqueous/membrane interface (xy plane). The surface tension value is 40 dyne/cm. All simulations started with a 5000-step energy minimization using a hybrid steepest descent/conjugated gradient algorithm. After minimization, each system was gradually heated from 0 to 310 K in 2 ns. Before pro-duction runs, all systems were equilibrated for 10 ns. Finally, 500 ns long productive MD simulations were carried out for data collection. The time step for the all-atom MD simulation was 2 fs.

Lipid order parameters. The calculation of lipid order parameters is theoretically analogous to the nuclear magnetic resonance spectroscopy of deuterated lipid chains. In MD simulations, the order parameters can be computed by the following equation:

$$S_{CD}=\frac{1}{2}(3\langle {cos}^{2}\left(\theta \right)\rangle -1)$$

where *θ* is the angle formed between the carbon–hydrogen bond vector and the *z*-axis (normal to the monolayer plane). *S_CD_* can range from −0.5 to 1. *S_CD_* tending to 1 indicates the tail is perfectly perpendicular to the monolayer plane, whereas *S_CD_* tending to 0 suggests complete randomness. In the limit value of −0.5, tails are oriented parallel to the plane.

## 4. Conclusions

In conclusion, here we developed a novel conjugation method to enhance the drug delivery effect for the disulfide units based on dynamic covalent disulfide exchange chemistry, especially for those that are less reactive, such as open-loop relaxed type disulfide units. In the case of the tert-butyl substituted disulfide unit, we synthesized the trigonometric bundling disulfide unit starship based on the 3,4,5-trihydroxyphenyl skeleton. Cell imaging experiments showed that this trigonometric bundling disulfide unit starship synergizes more effectively to promote cellular uptake more quickly, and it completely compensates for the disadvantage regarding the low reactivity of the *tert*-butyl substituted disulfide unit. Furthermore, studies on thiol inhibition of the cell membrane revealed that this starship is also an endocytosis-independent internalization mechanism. Therefore, this disulfide unit starship has the potential to be an effective drug delivery agent. Research is currently underway into the delivery of macromolecular nucleic acids.

## Data Availability

The raw data supporting the conclusion of this article will be made available by the authors on request.

## Supplementary Materials

The supporting information can be downloaded at https://www.mdpi.com/article/10.3390/ijms25147518/s1.
