# Supplementary material for: Trigonometric Bundling Disulfide Unit Starship Synergizes More Effectively to Promote Cellular Uptake
Source: Int J Mol Sci. 2024 Jul 9;25(14):7518. doi: 10.3390/ijms25147518 (PMC11277142; doi:10.3390/ijms25147518)
Supplement: Supplementary file 1 [file ijms-25-07518-s001.zip › ijms-3042787-supplementary.pdf]

Table of contents

Figure S1. $^1H/^{13}C$ NMR and HRMS spectra of compound **2** 3
Figure S2. $^1H/^{13}C$ NMR and HRMS spectra of compound **5a**. 4
Figure S3. $^1H/^{13}C$ NMR and HRMS spectra of compound **5b**. 6
Figure S4. $^1H/^{13}C$ NMR and HRMS spectra of compound **6a**. 6
Figure S5. ^1H NMR and HRMS spectra of compound **6b'**. 7
Figure S6. $^1H/^{13}C$ NMR and HRMS spectra of compound **6b**. 9
Figure S7. $^1H/^{13}C$ NMR and HRMS spectra of compound **7a'**. 10
Figure S8. $^1H/^{13}C$ NMR and HRMS spectra of compound **7a**. 12
Figure S9. $^1H/^{13}C$ NMR and HRMS spectra of compound **7b'**. 13
Figure S10. $^1H/^{13}C$ NMR and HRMS spectra of compound **7b**. 15
Figure S11. $^1H/^{13}C$ NMR and HRMS spectra of compound **8a**. 16
Figure S12. $^1H/^{13}C$ NMR and HRMS spectra of compound **8b**. 18
Figure S13. CLSM images of A549 cells after incubation with 1 μM FITC-NH_2 probe together with DAPI to stain the nuclei. 19
Figure S14. CLSM images of HeLa S3 cells after incubation with 1 μM **SS1/SS3-FITC** probe together with DAPI to stain the nuclei. 19
Figure S15. CLSM images of MDA-MB-231 (A and B) and HepG2 (C and D) cells after incubation with 1 μM **SS1/SS3-FITC** probe 4hr together with DAPI to stain the nuclei. 20
Figure S16. CLSM images of HT29 (A and B) and MCF-7 (C and D) cells after incubation with 1 μM **SS1/SS3-FITC** probe 4hr together with DAPI to stain the nuclei. 21
Figure S17. Quantitative analysis of fluorescence intensity using SS1/3-FITC in various cell lines. 21
Figure S18. Energy profile for different disulfide unit. 22

N20230209-FC0006-WL1.1.fid —

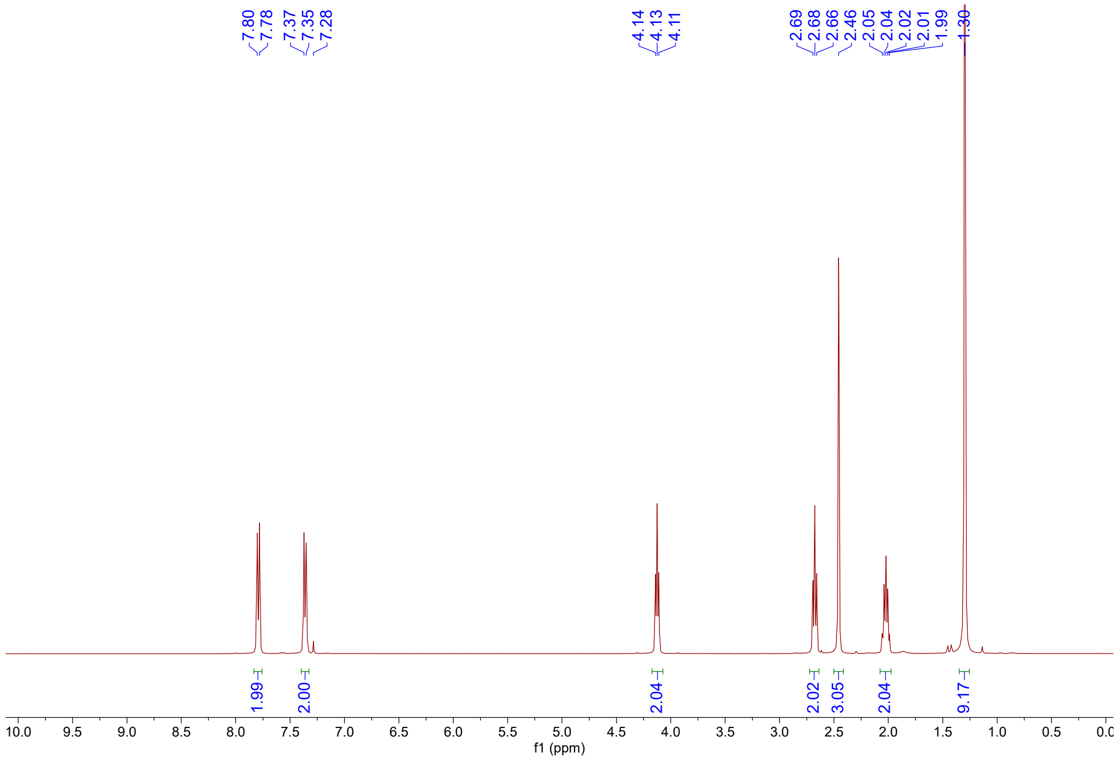

N20230209-FC0006-WL1.2.fid —

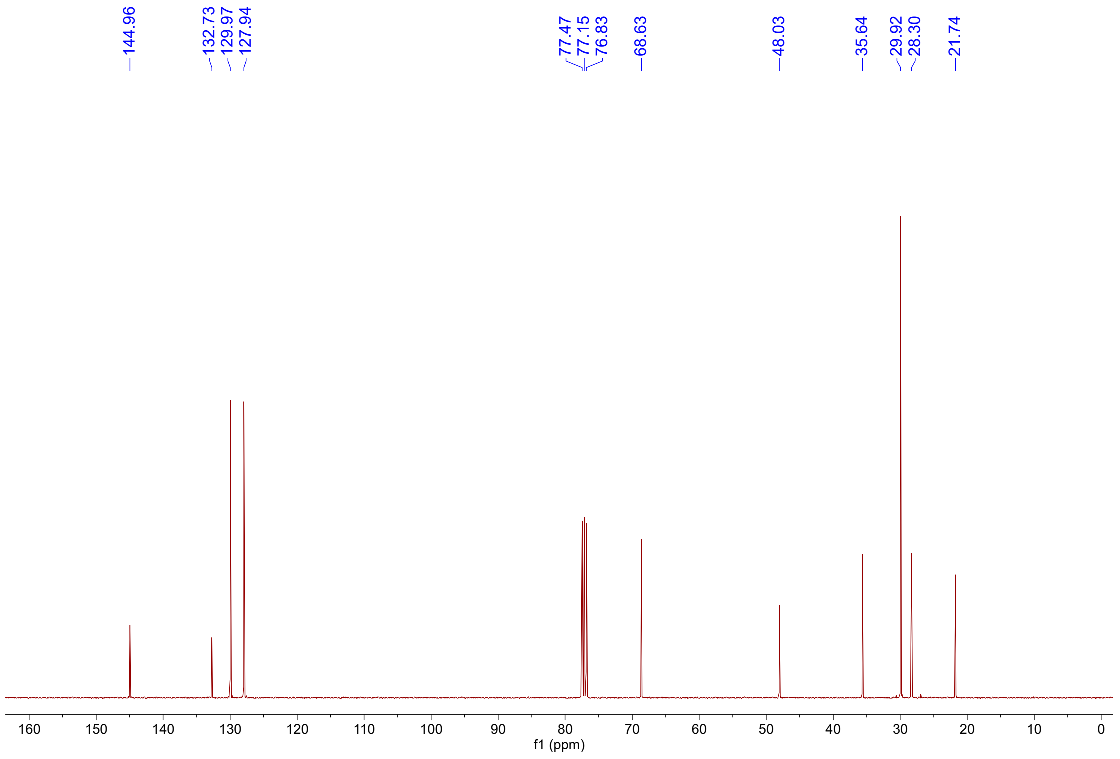

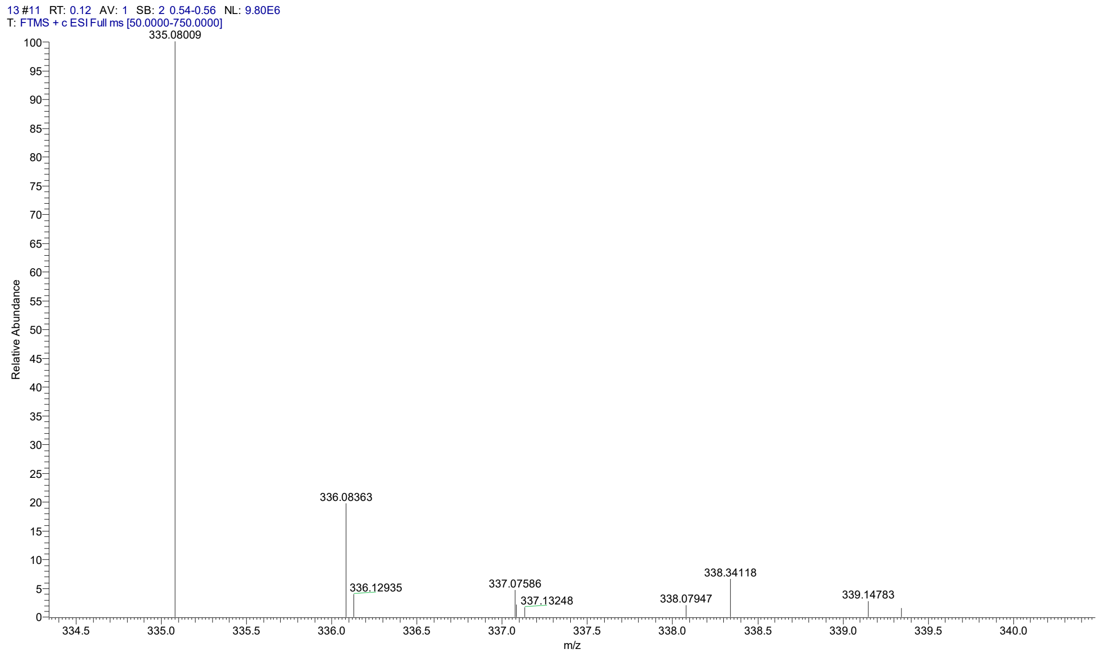

Figure S1. $^{1}H/^{13}C$ NMR and HRMS spectra of compound **2**.

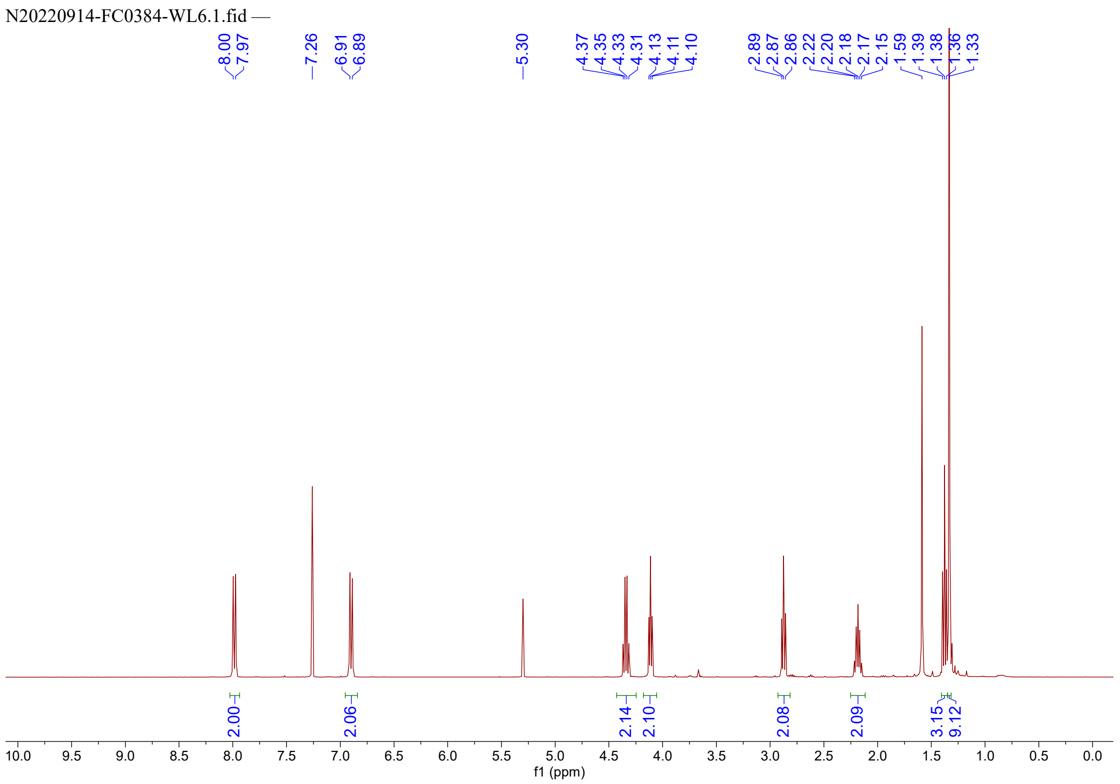

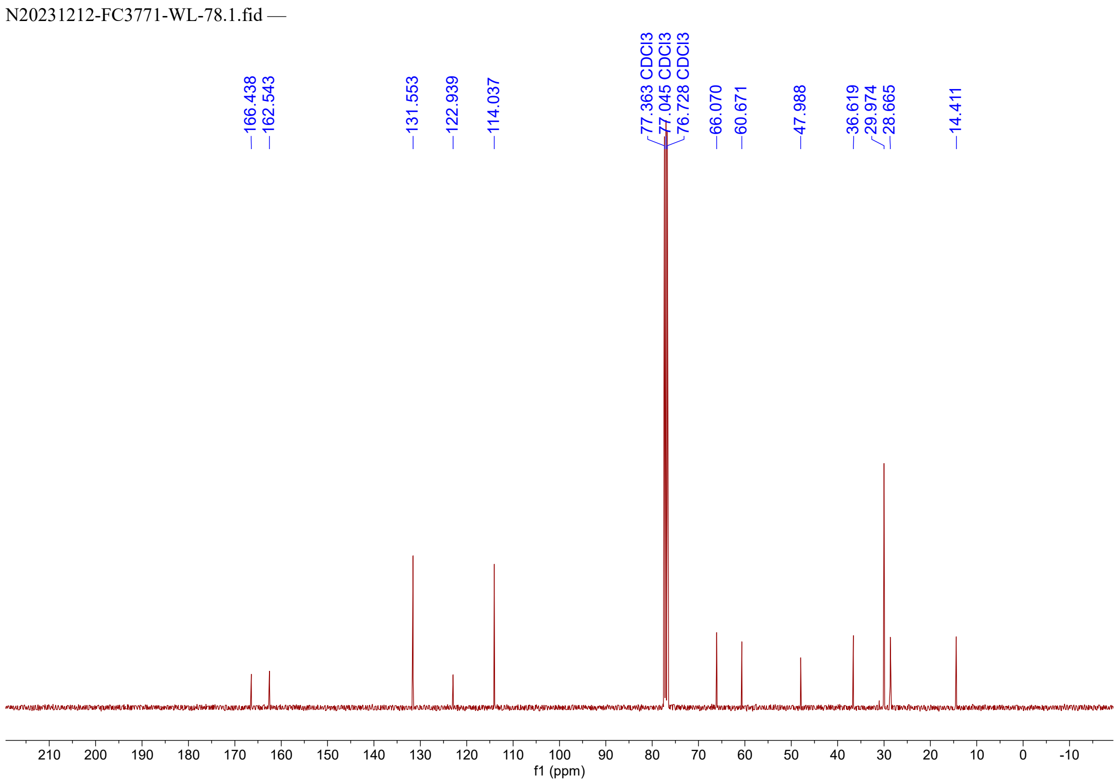

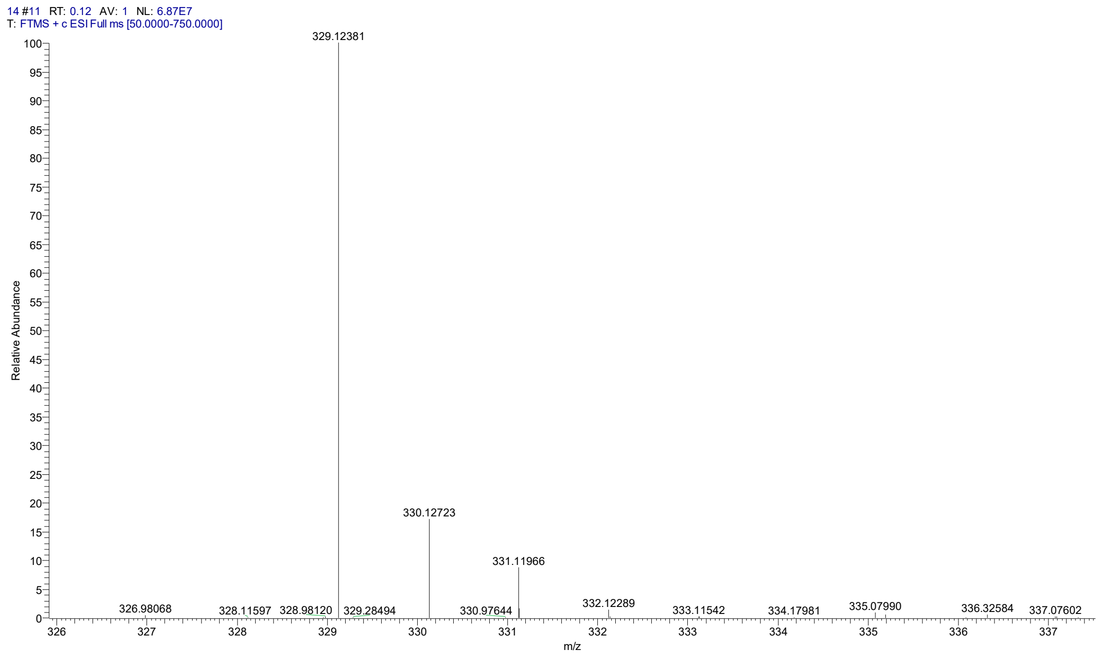

Figure S2. $^{1}H/^{13}C$ NMR and HRMS spectra of compound **5a**.

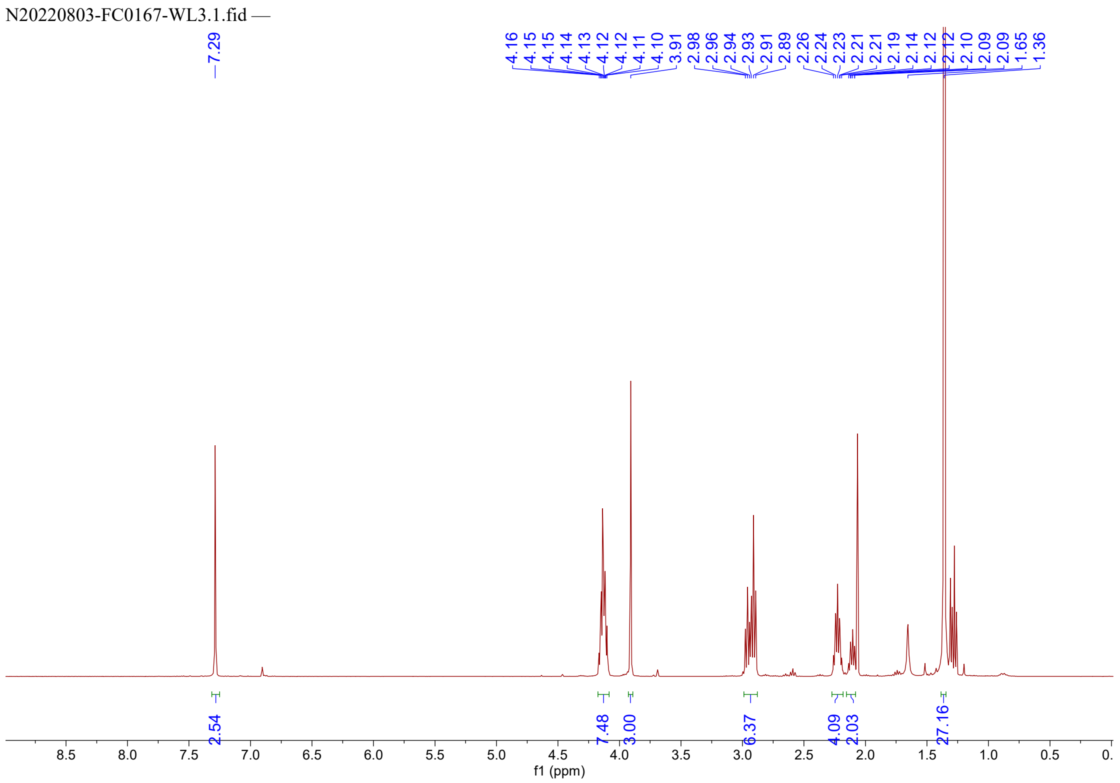
N20220803-FC0167-WL3.1.fid —
7.29
4.16
4.15
4.15
4.14
4.13
4.12
4.12
4.11
4.10
3.91
2.98
2.96
2.94
2.93
2.91
2.89
2.26
2.24
2.23
2.21
2.21
2.19
2.14
2.12
2.12
2.10
2.09
2.09
1.65
1.36
2.54
7.48
3.00
6.37
4.09
2.03
27.16
f1 (ppm)

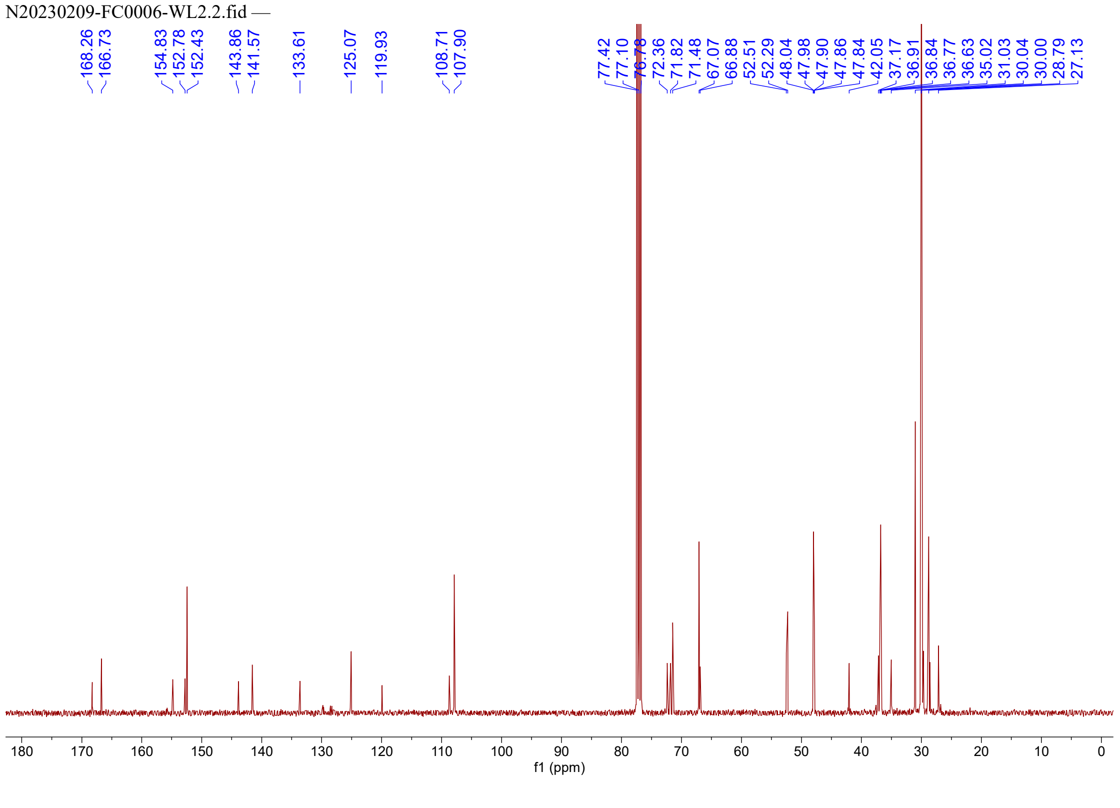
N20230209-FC0006-WL2.2.fid —
168.26
166.73
154.83
152.78
152.43
143.86
141.57
133.61
125.07
119.93
108.71
107.90
77.42
77.10
76.78
72.36
71.82
71.48
67.07
66.88
52.51
52.29
48.04
47.98
47.90
47.86
47.84
42.05
37.17
36.91
36.84
36.77
36.63
35.02
31.03
30.04
30.00
28.79
27.13
f1 (ppm)

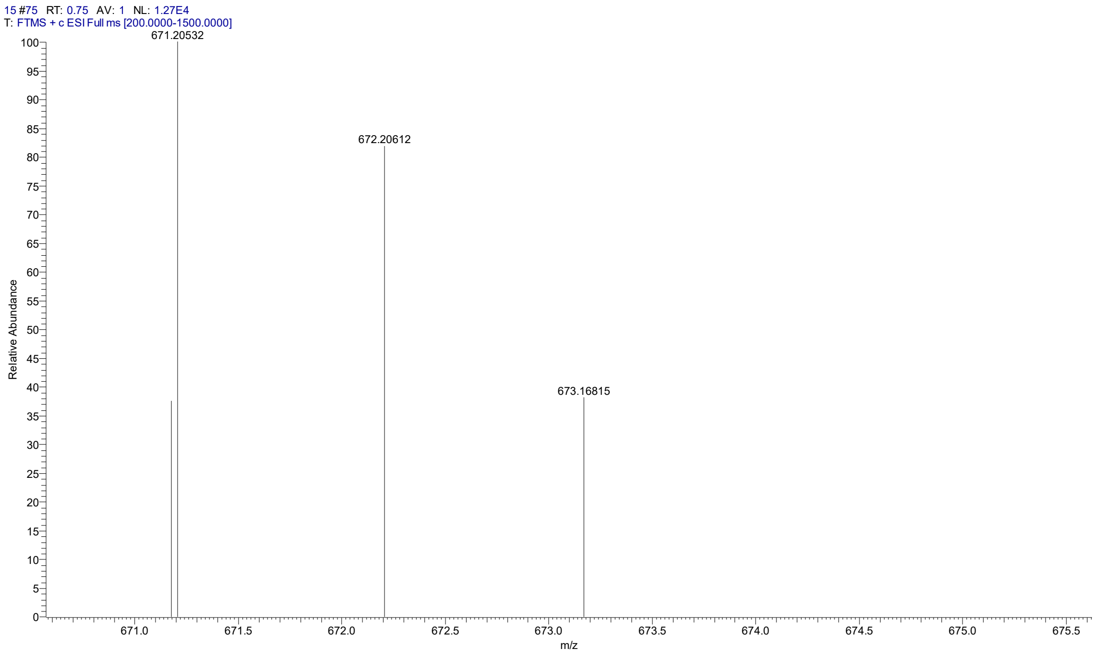

Figure S3. $^{1}H/^{13}C$ NMR and HRMS spectra of compound **5b**.

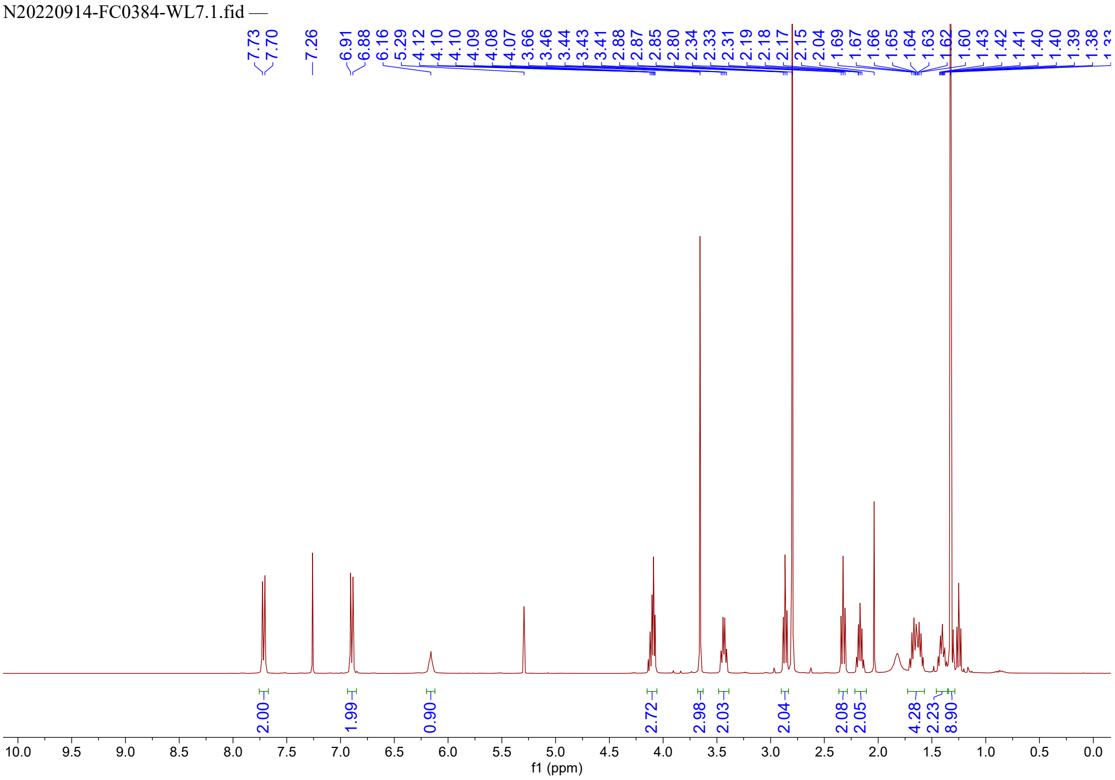

Figure S4. $^{1}H/^{13}C$ NMR and HRMS spectra of compound **6a**.

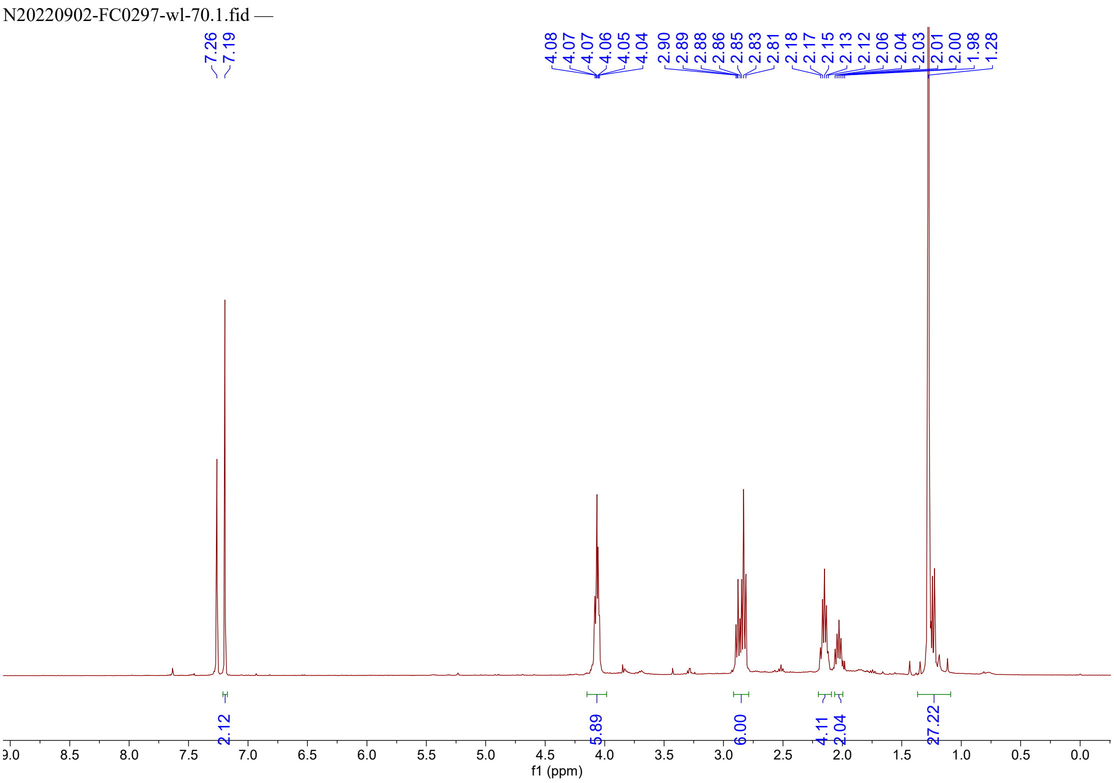

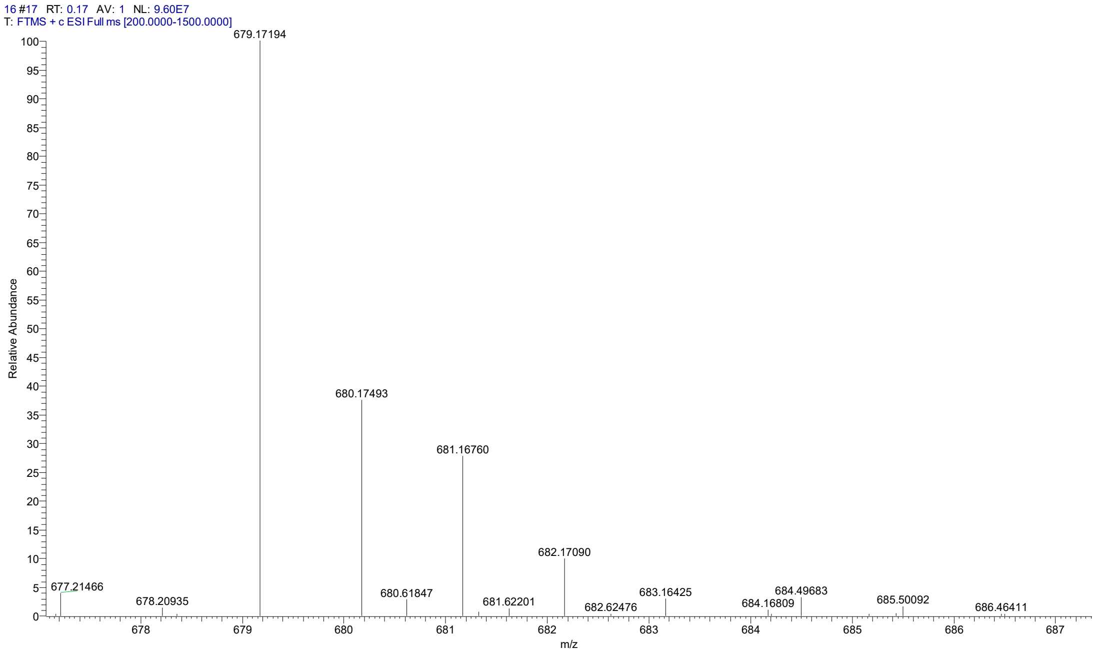

Figure S5. ^{1}H NMR and HRMS spectra of compound **6b’**.

N20220914-FC0384-WL4.1.fid —

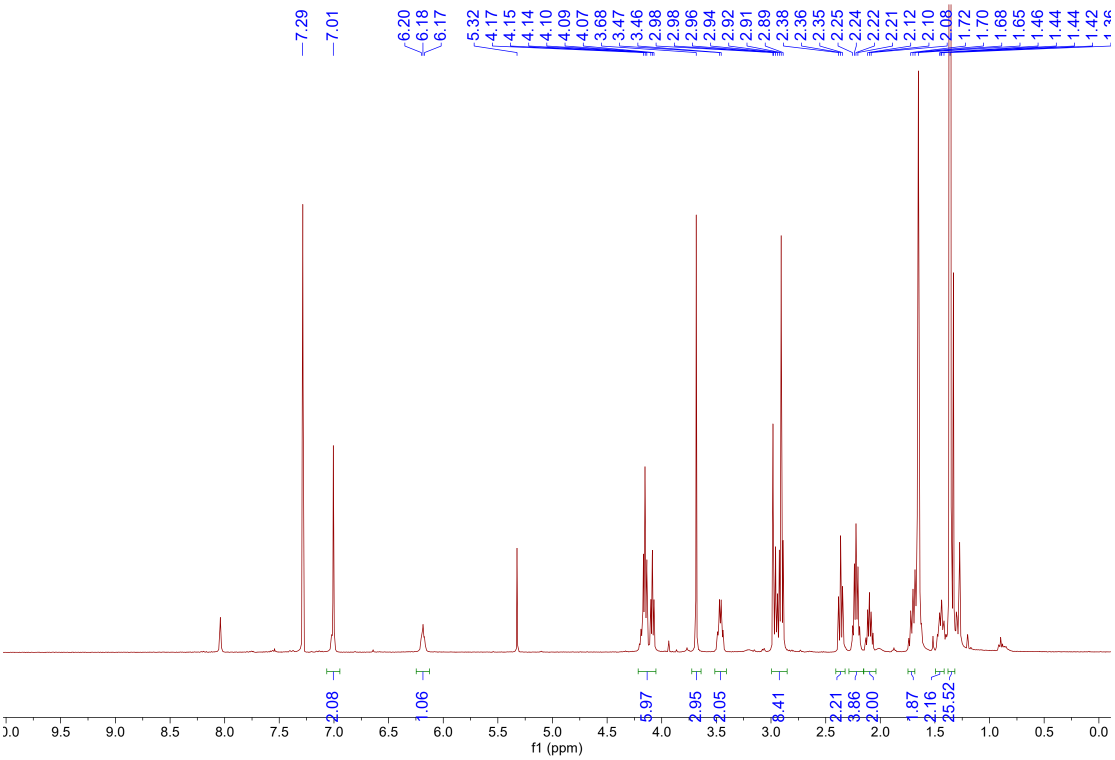

N20231212-FC3771-WL-74.1.fid —

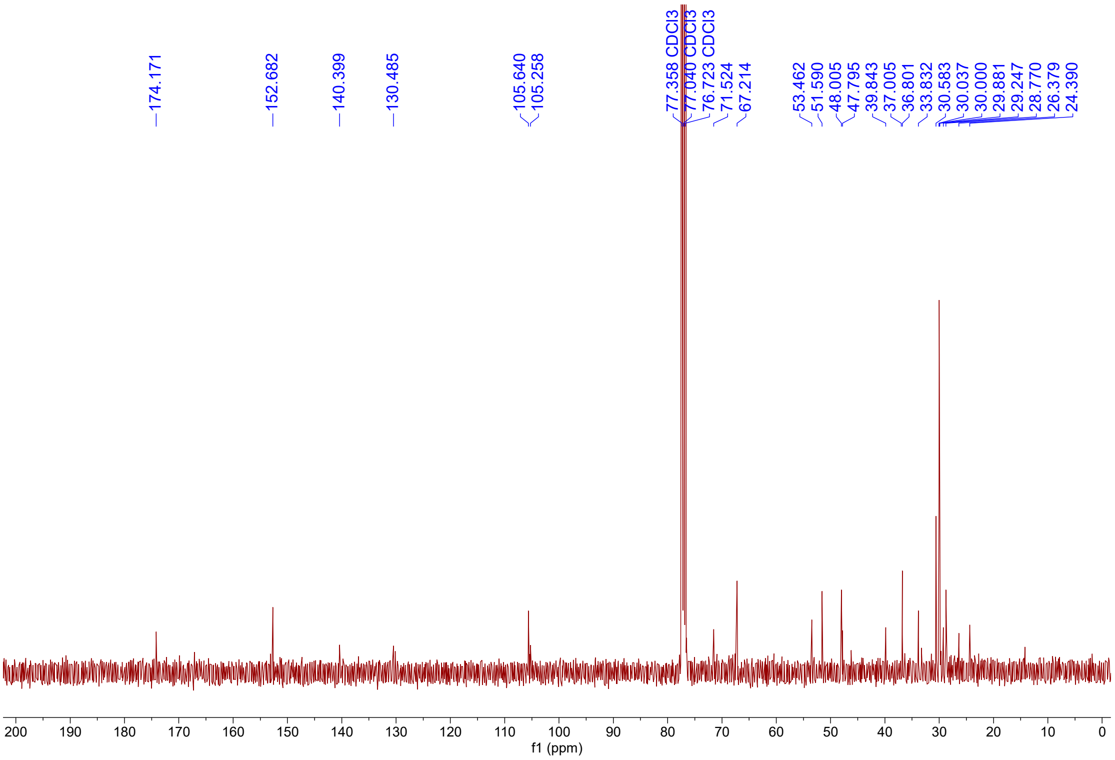

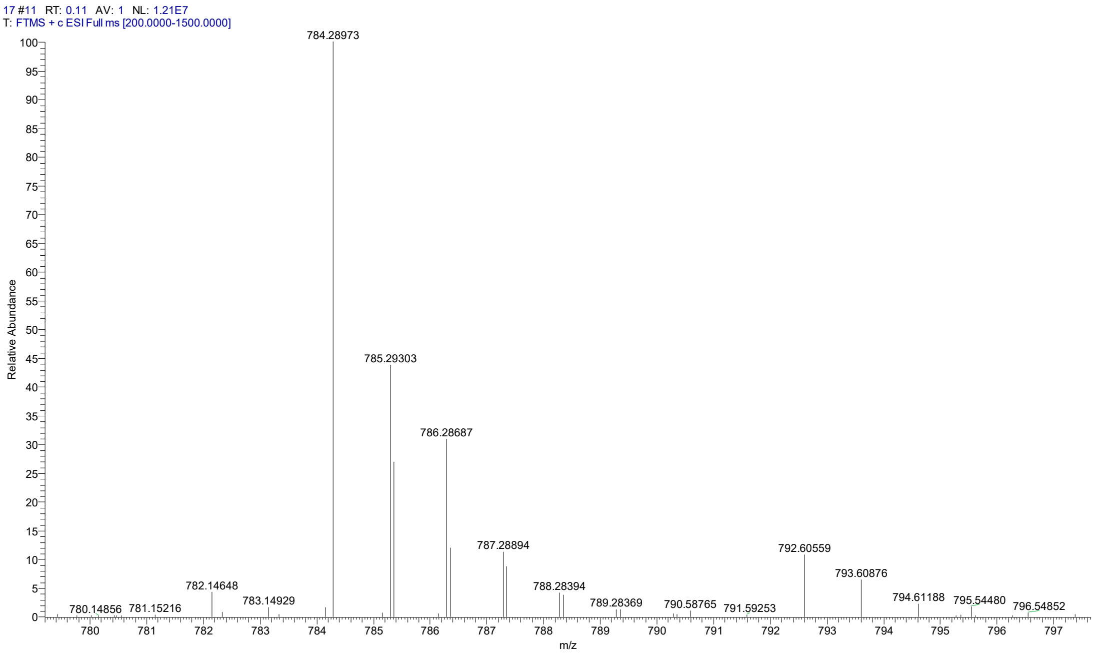

Figure S6. $^{1}H/^{13}C$ NMR and HRMS spectra of compound **6b**.

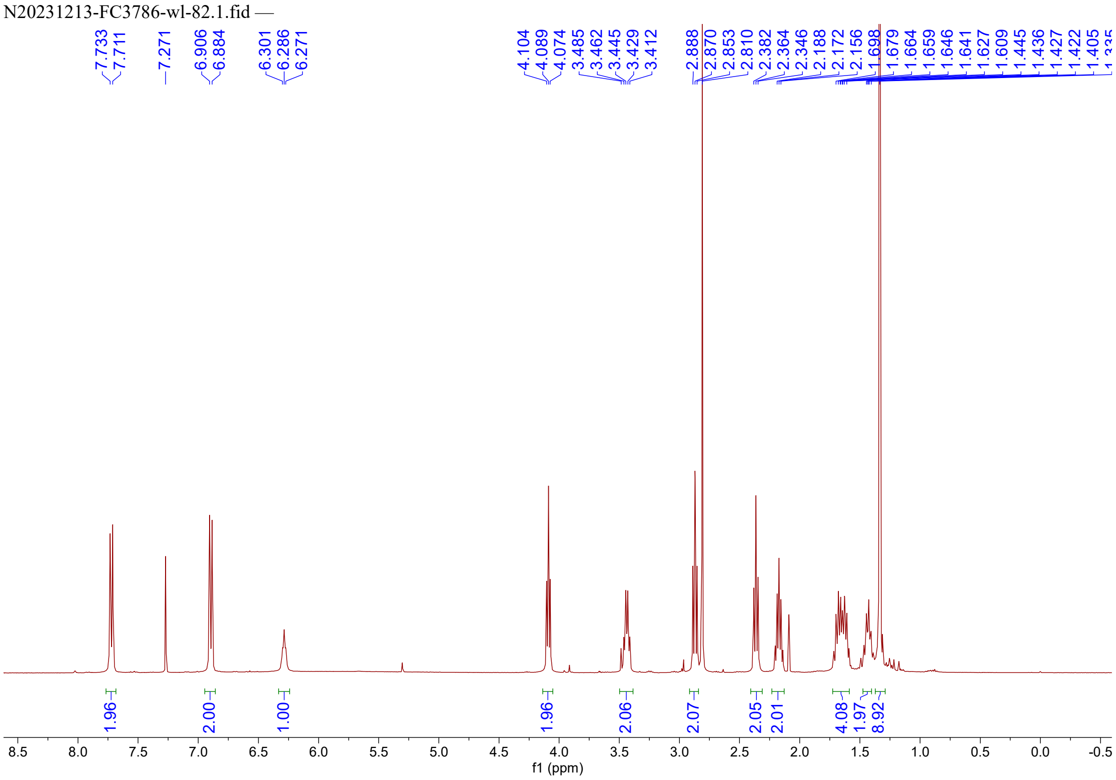

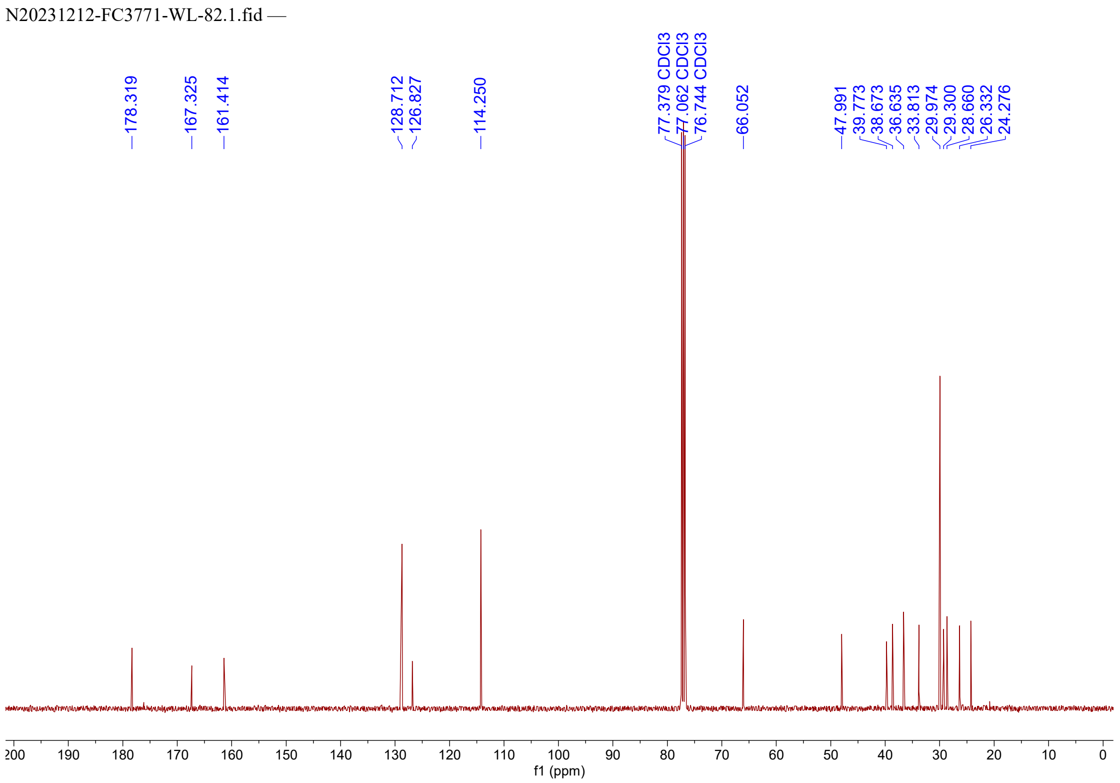

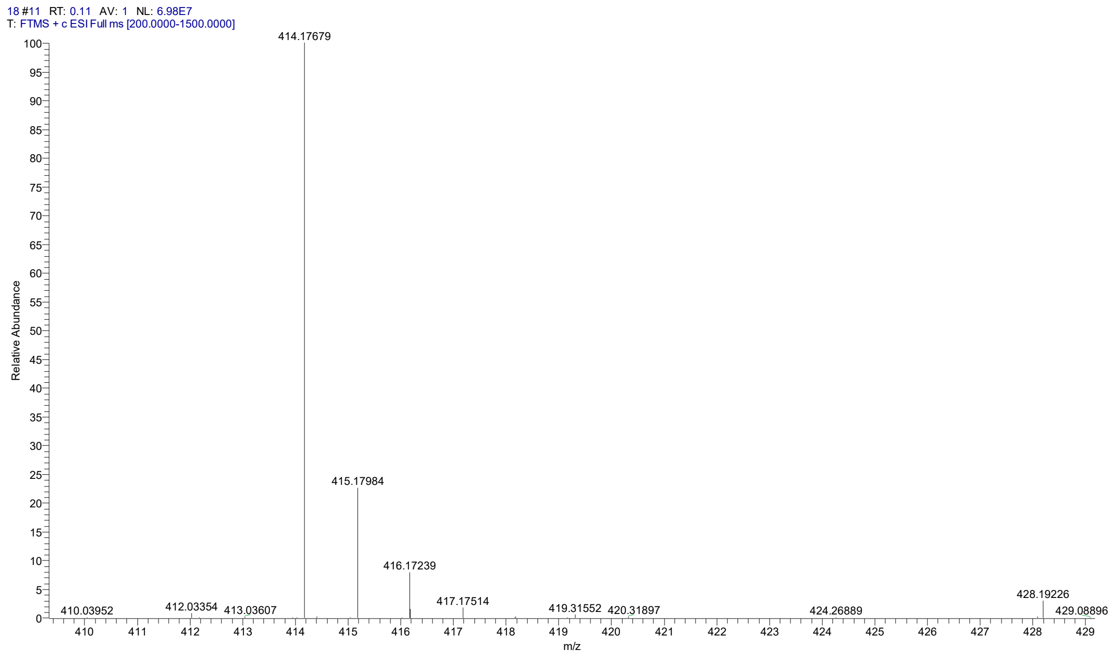

Figure S7. $^{1}H/^{13}C$ NMR and HRMS spectra of compound **7a’**.

N20230407-FC0725-wl-170.1.fid —

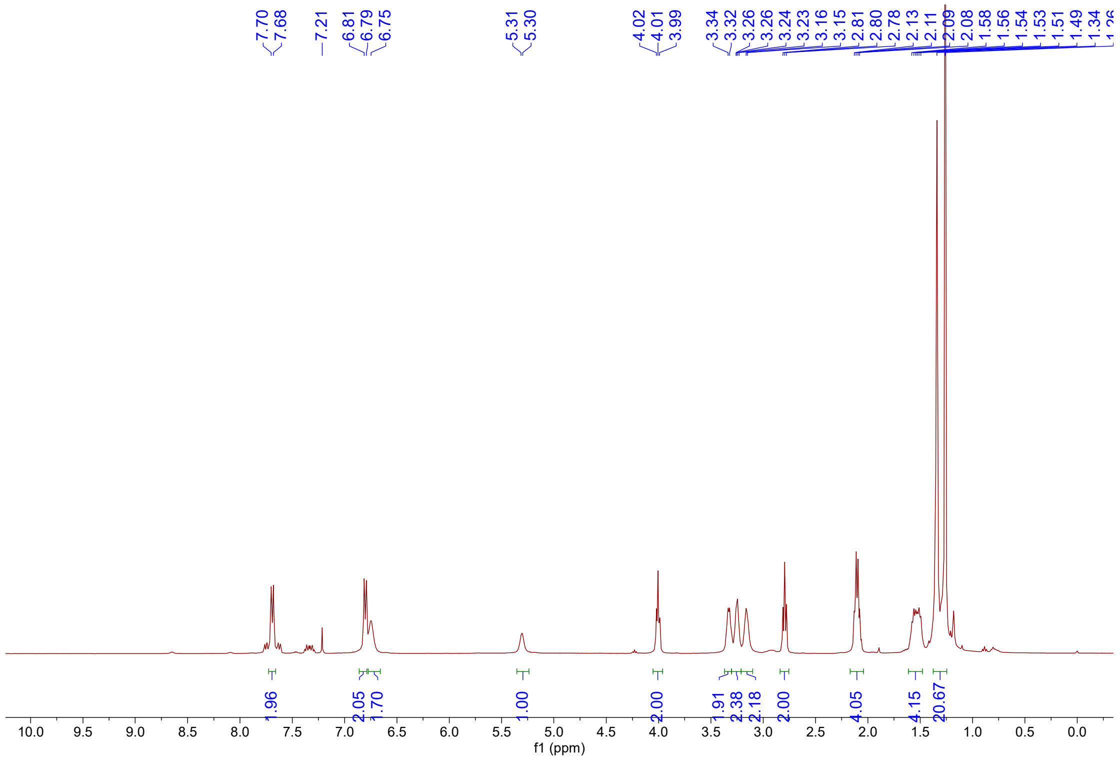

N20230407-FC0725-wl-170.2.fid —

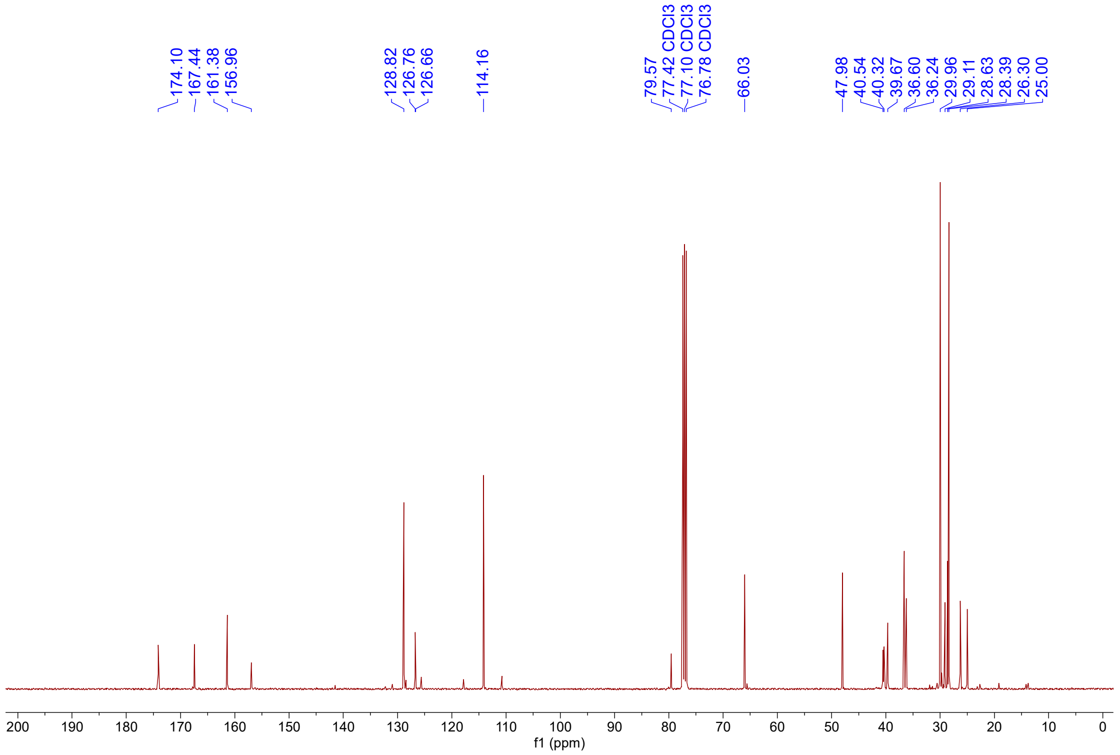

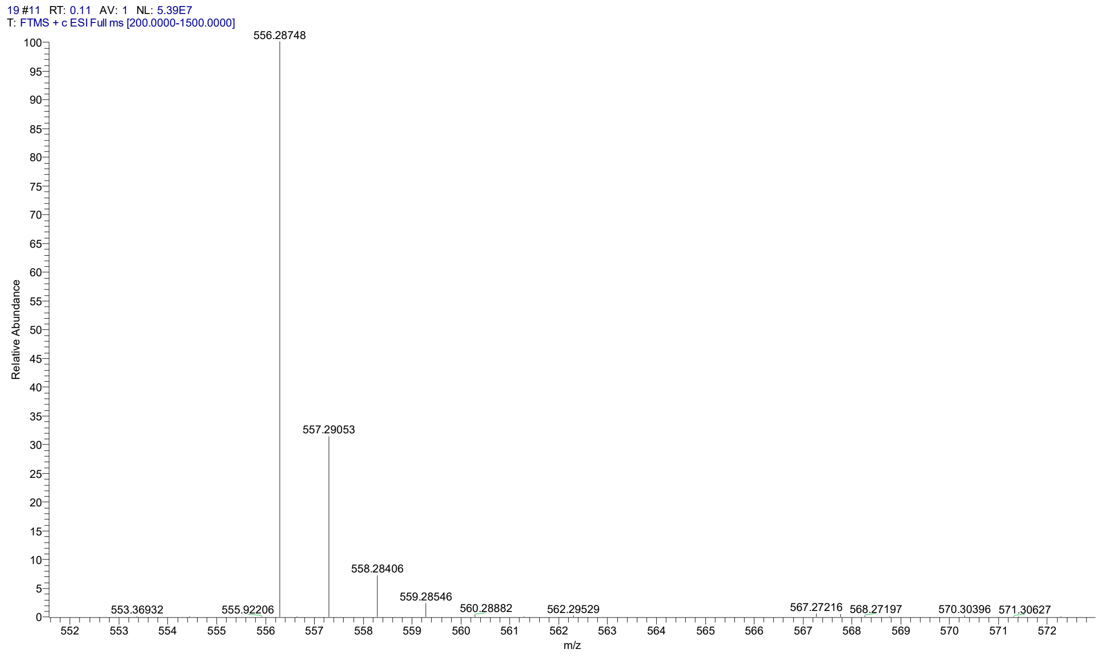

Figure S8. $^{1}H/^{13}C$ NMR and HRMS spectra of compound **7a**.

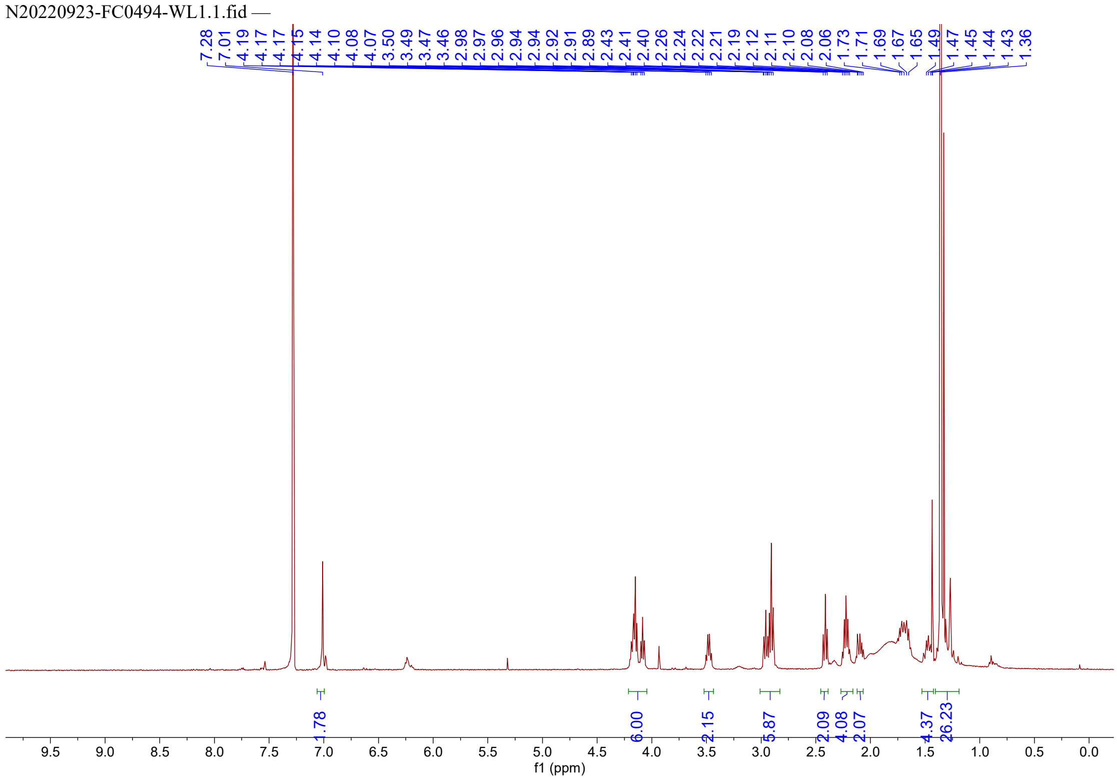

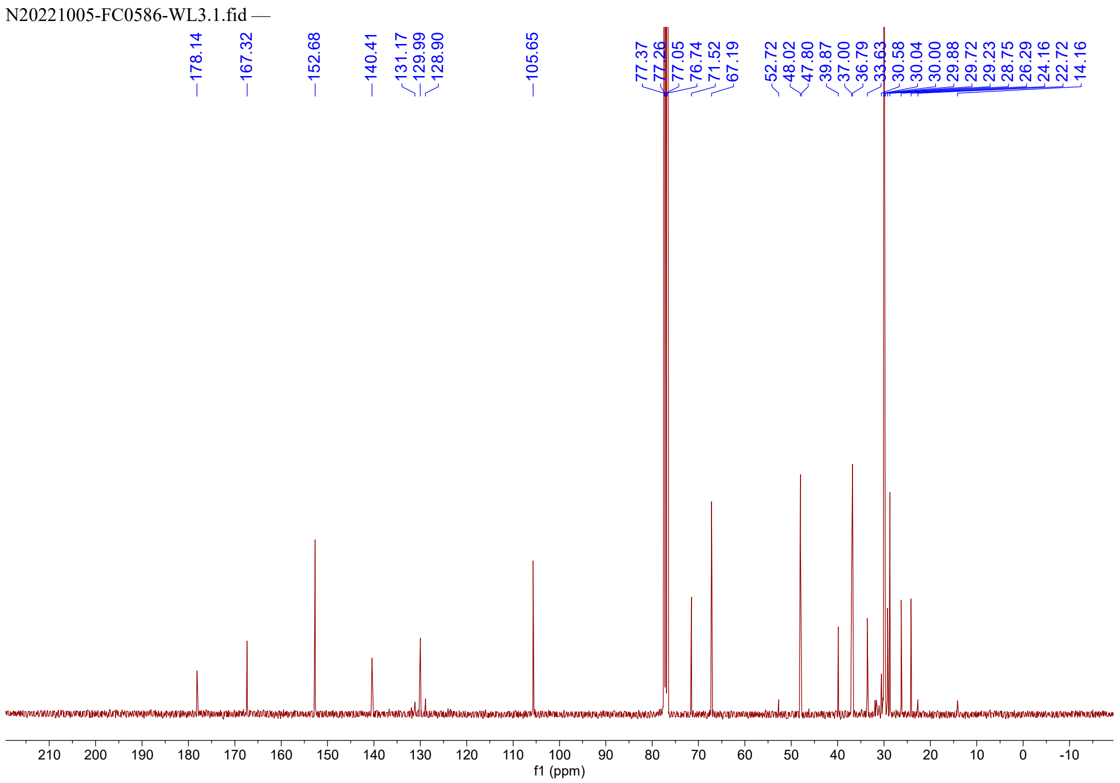

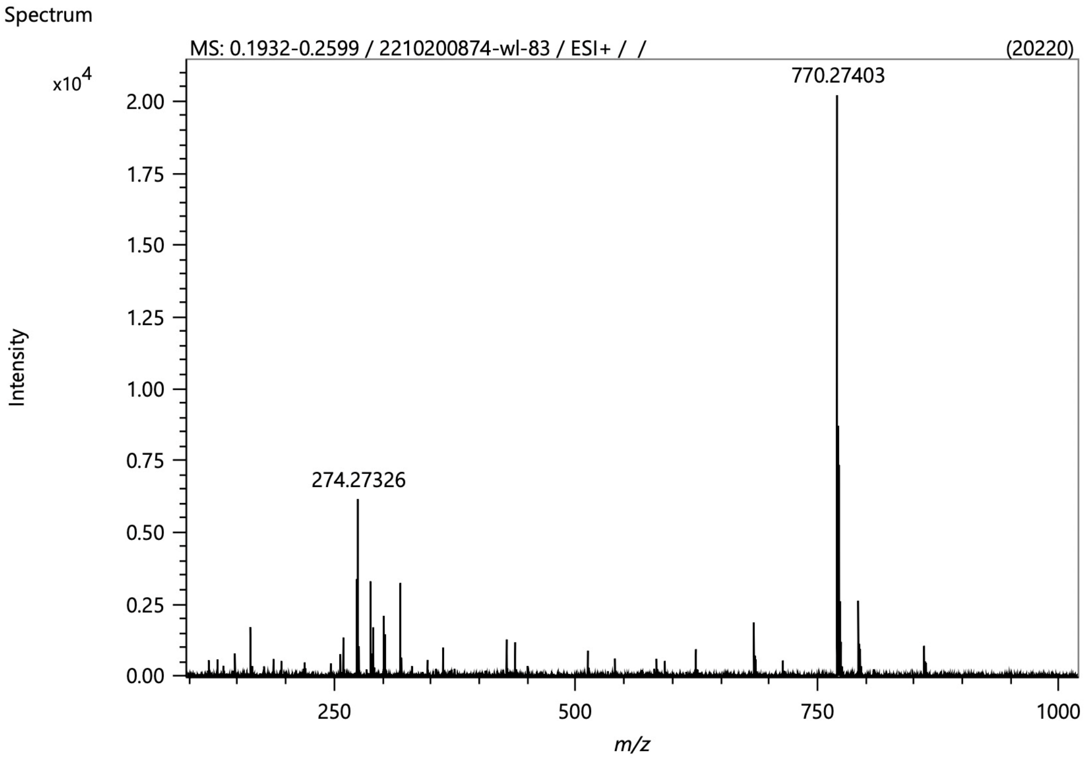

Figure S9. $^{1}H/^{13}C$ NMR and HRMS spectra of compound **7b’**.

N20230412-FC0809-wl-179.1.fid —

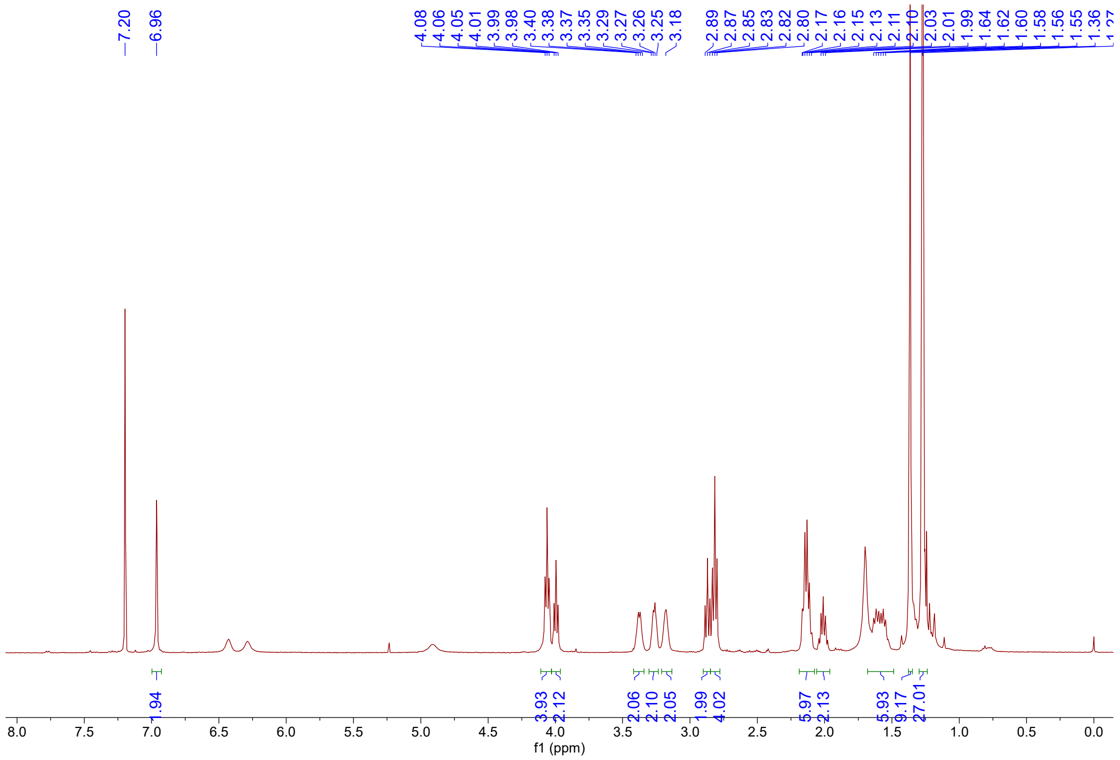

N20231212-FC3771-WL-179.1.fid —

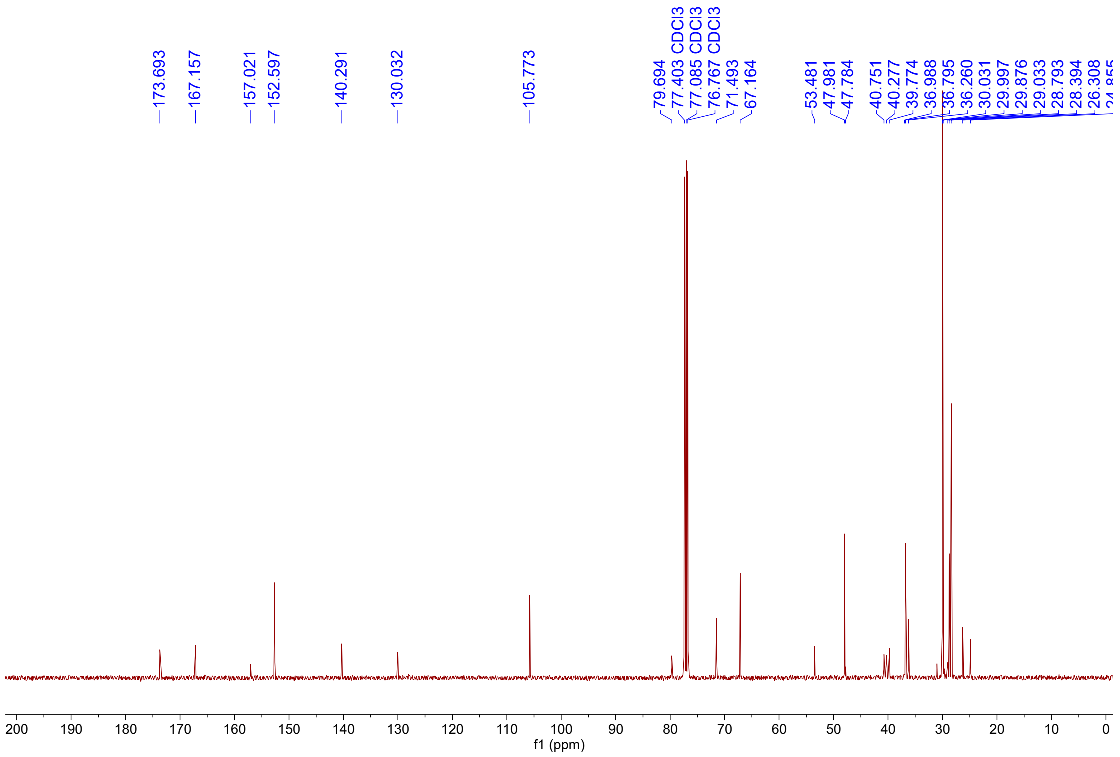

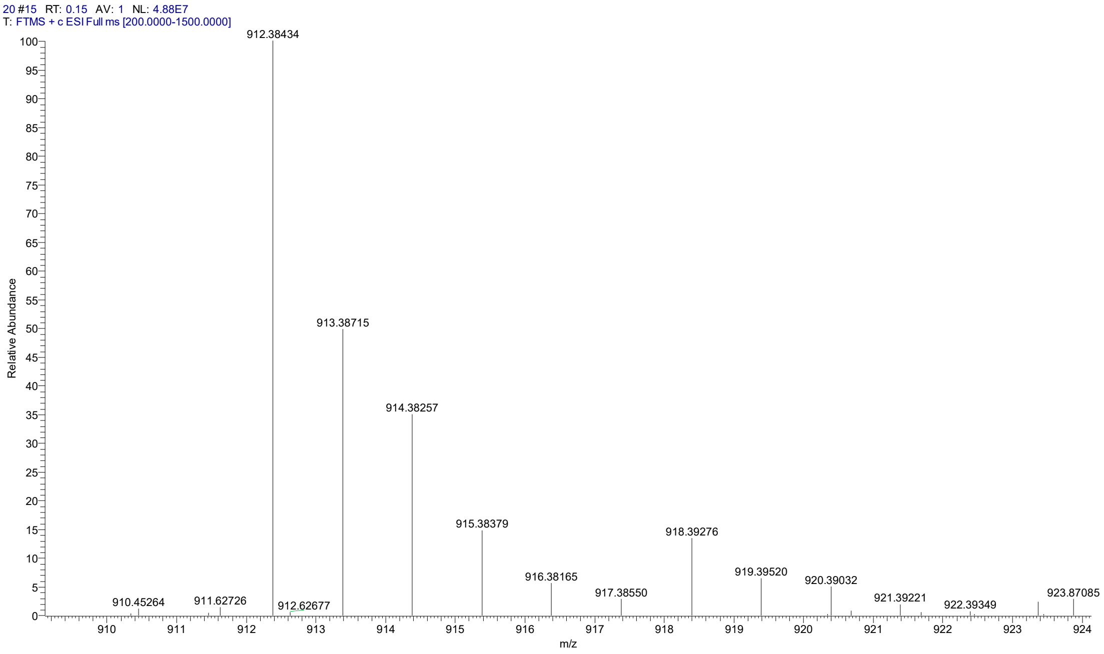

Figure S10. $^{1}H/^{13}C$ NMR and HRMS spectra of compound **7b**.

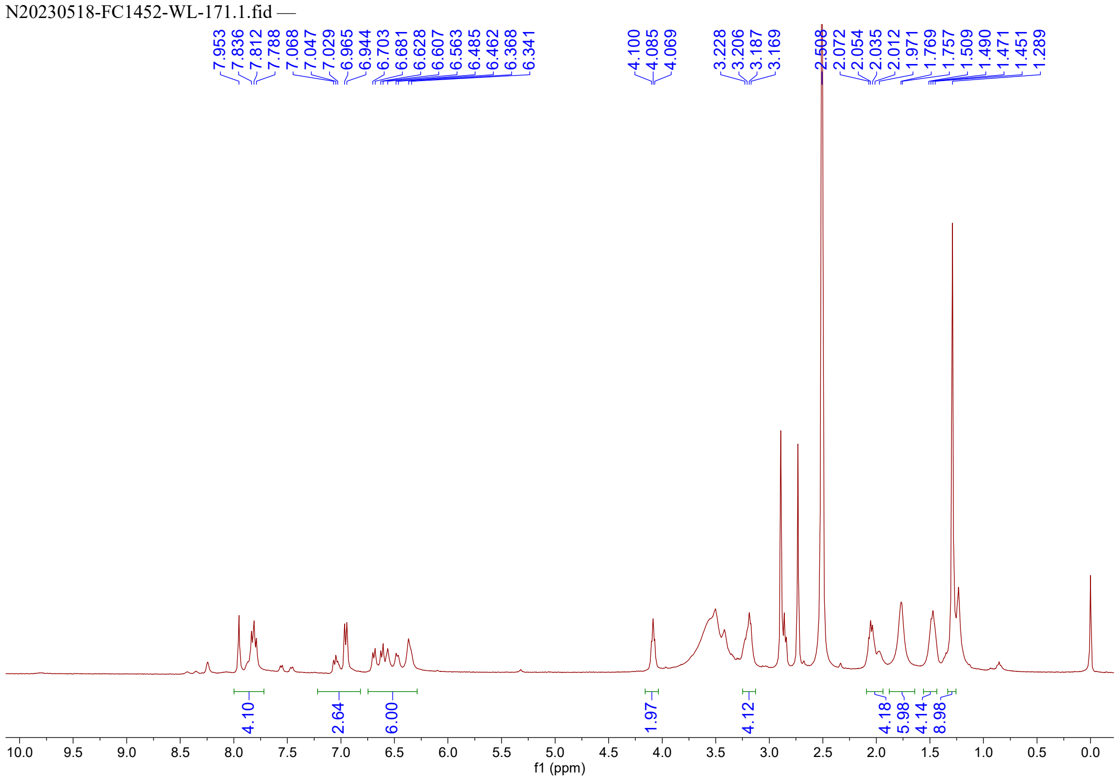

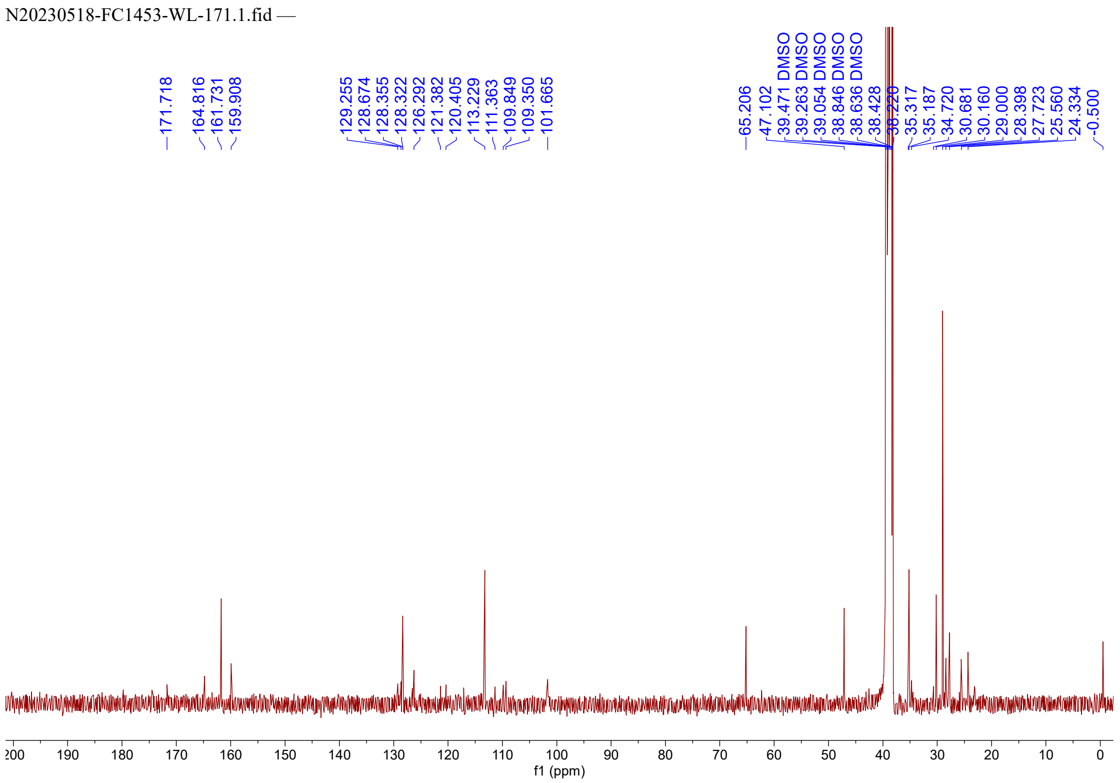

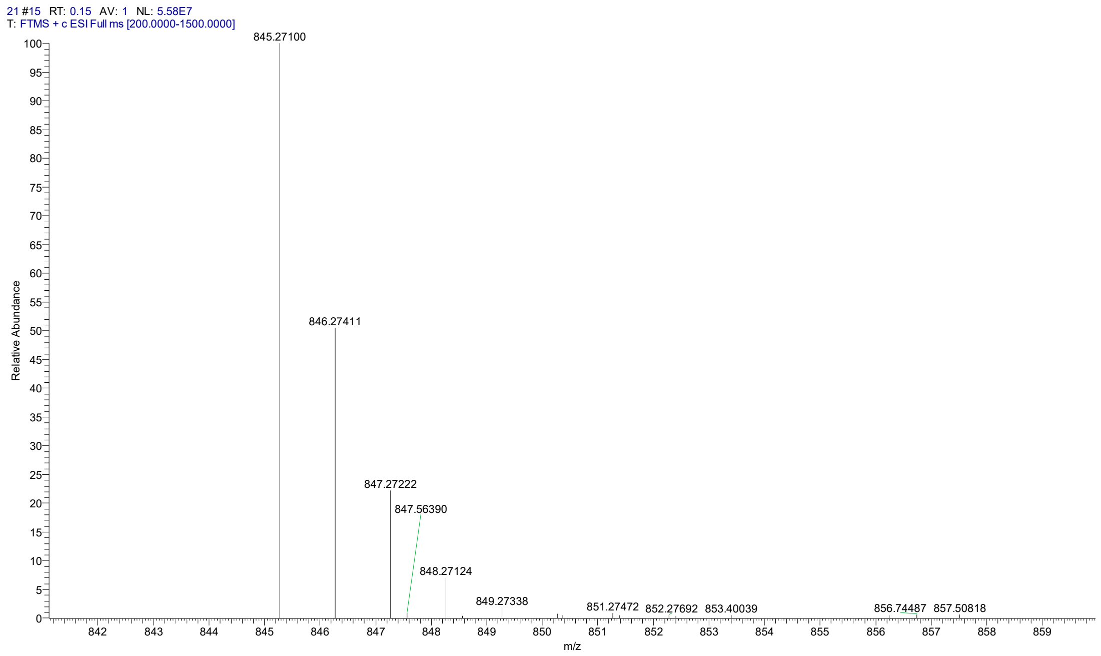

Figure S11. ^{1}H/^{13}C NMR and HRMS spectra of compound **8a**.

N20230511-FC1294-WL-180.1.fid —

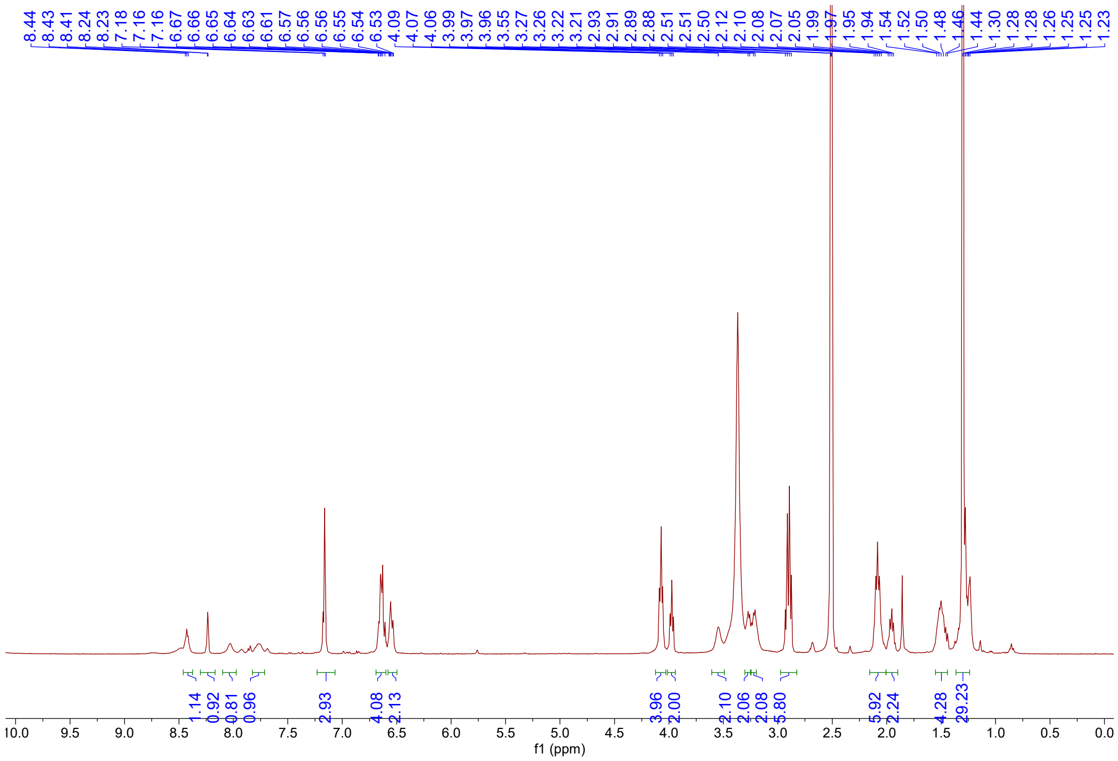

N20230511-FC1294-WL-180.2.fid —

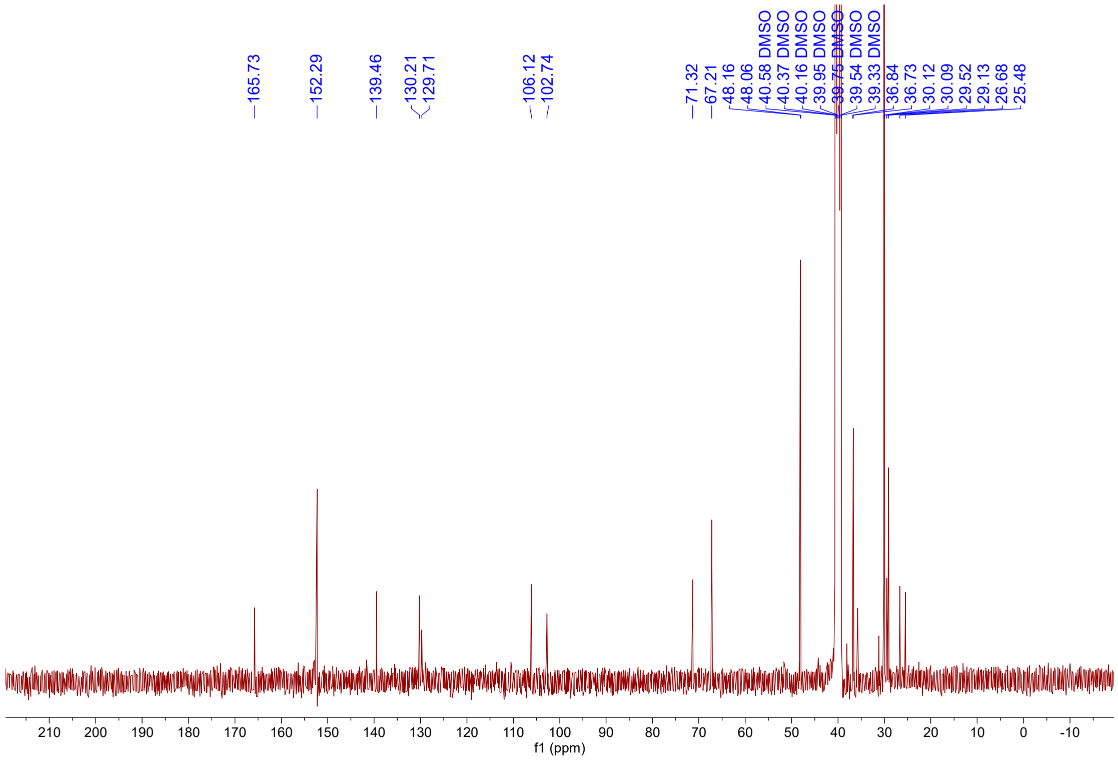

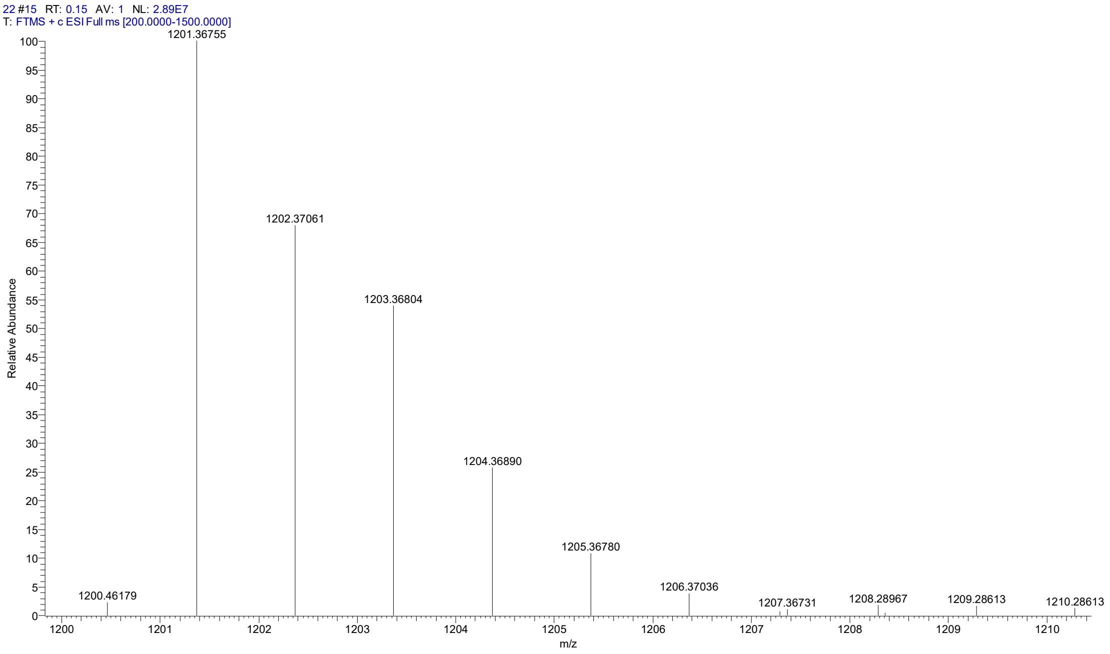

Figure S12. $^{1}H/^{13}C$ NMR and HRMS spectra of compound **8b**.

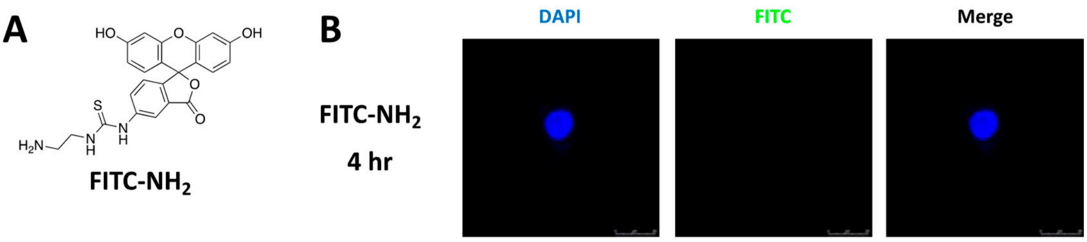

Figure S13. CLSM images of A549 cells after incubation with 1 μM FITC-NH_2 probe together with DAPI to stain the nuclei.

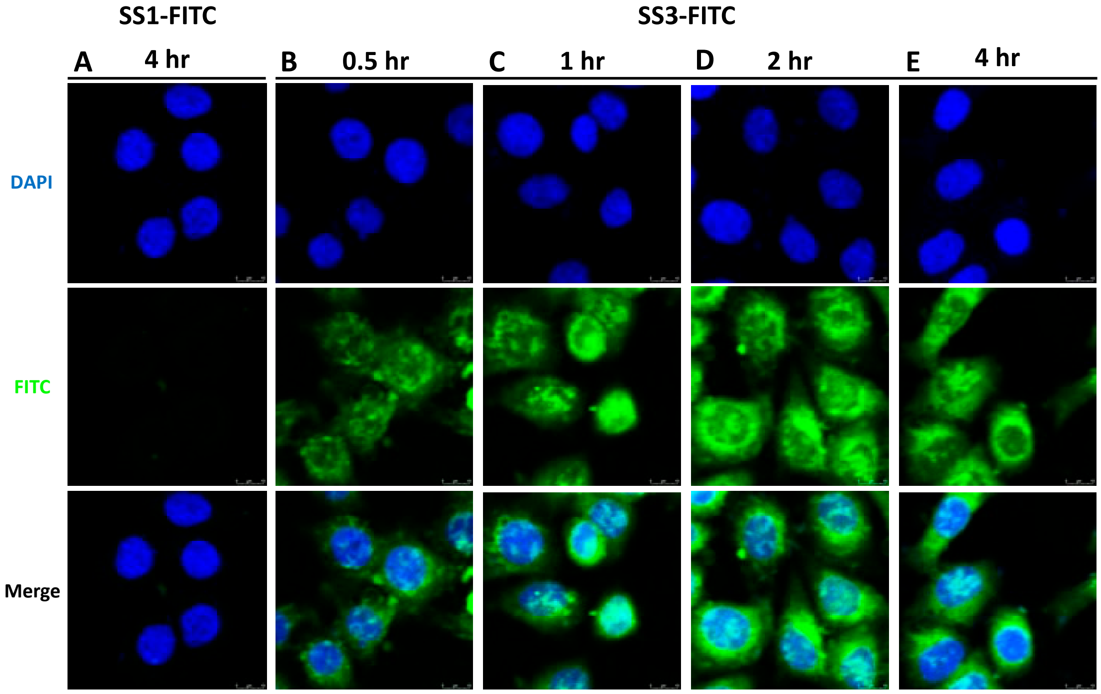

Figure S14. (A-E) CLSM images of HeLa S3 cells after incubation with 1 μM **SS1/SS3-FITC** probe together with DAPI to stain the nuclei.

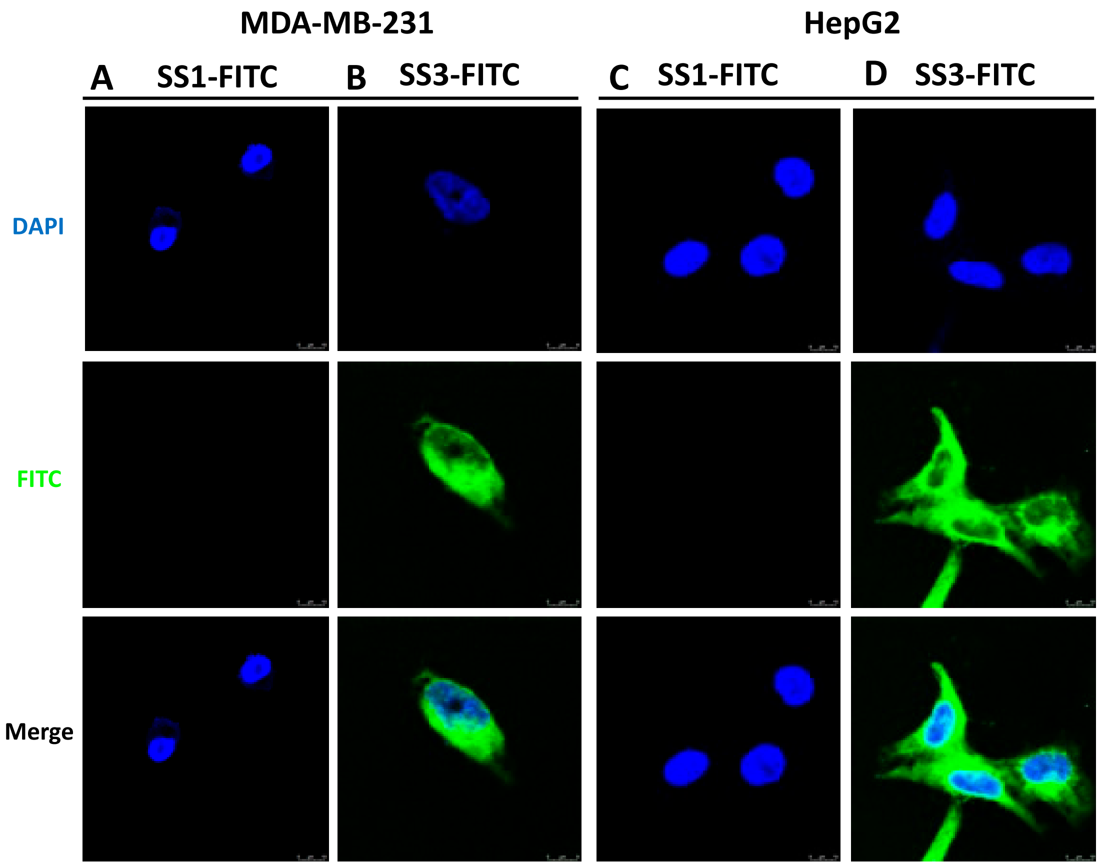

Figure S15. CLSM images of MDA-MB-231 (A and B) and HepG2 (C and D) cells after incubation with 1 μM **SS1/SS3-FITC** probe 4hr together with DAPI to stain the nuclei.

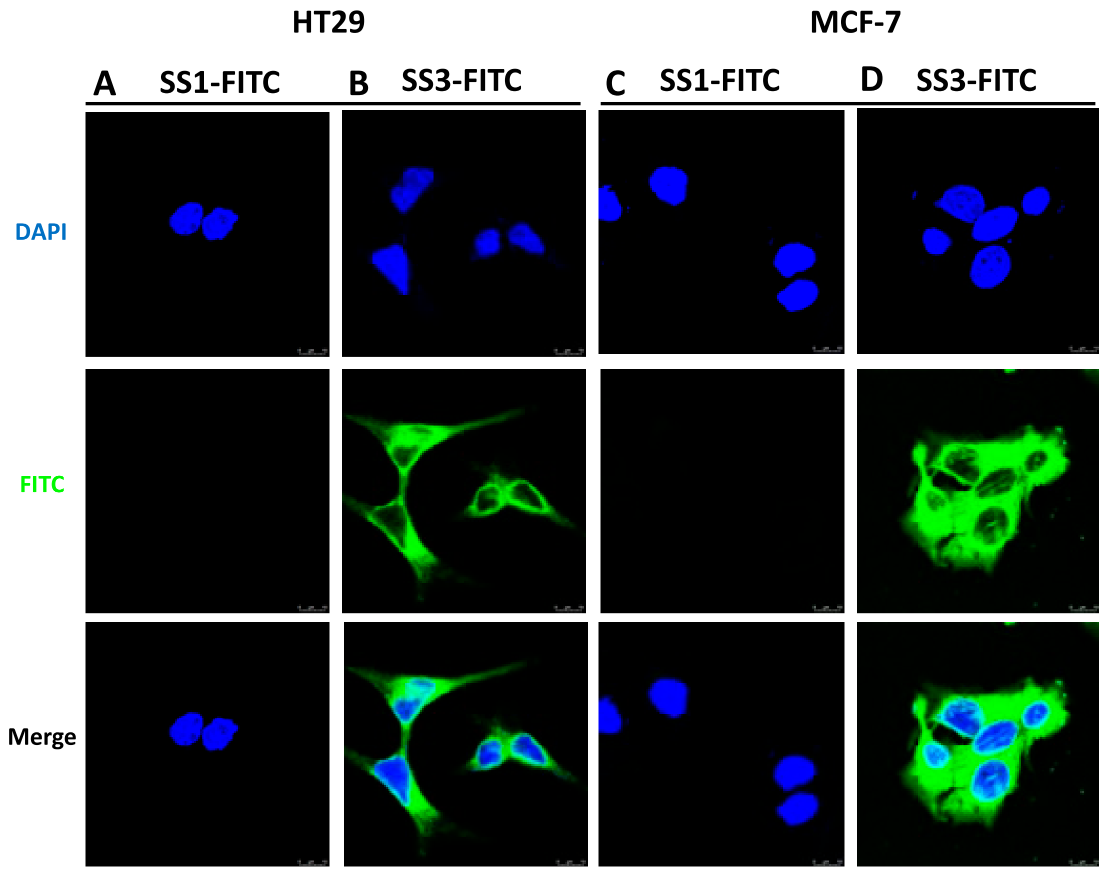

Figure S16. CLSM images of HT29 (A and B) and MCF-7 (C and D) cells after incubation with 1 μM **SS1/SS3-FITC** probe 4hr together with DAPI to stain the nuclei.

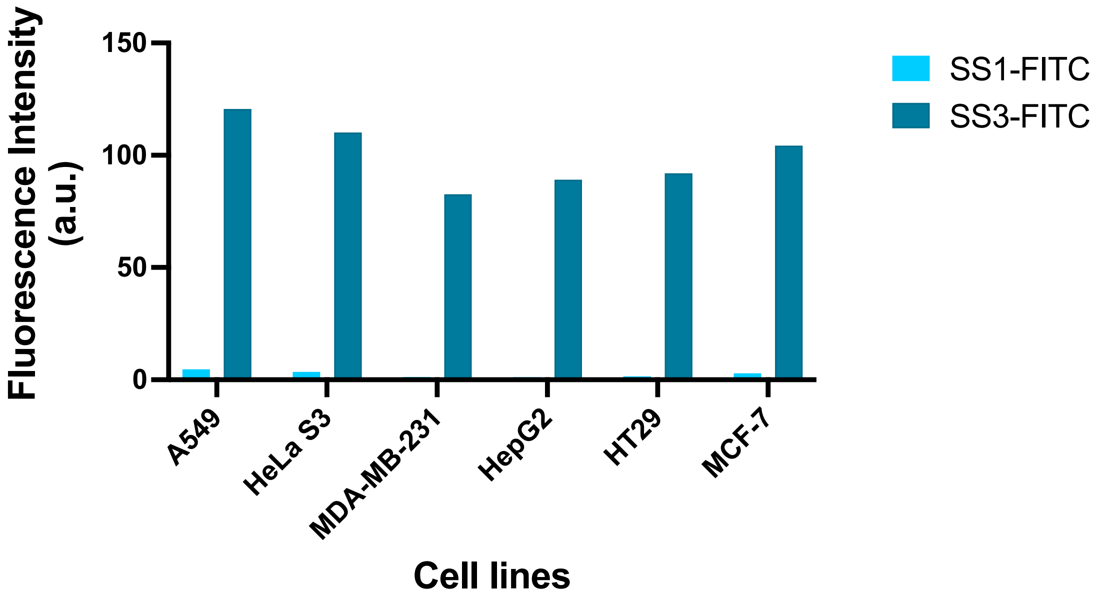

Figure S17. Quantitative analysis of fluorescence intensity using SS1/3-FITC in various cell lines.

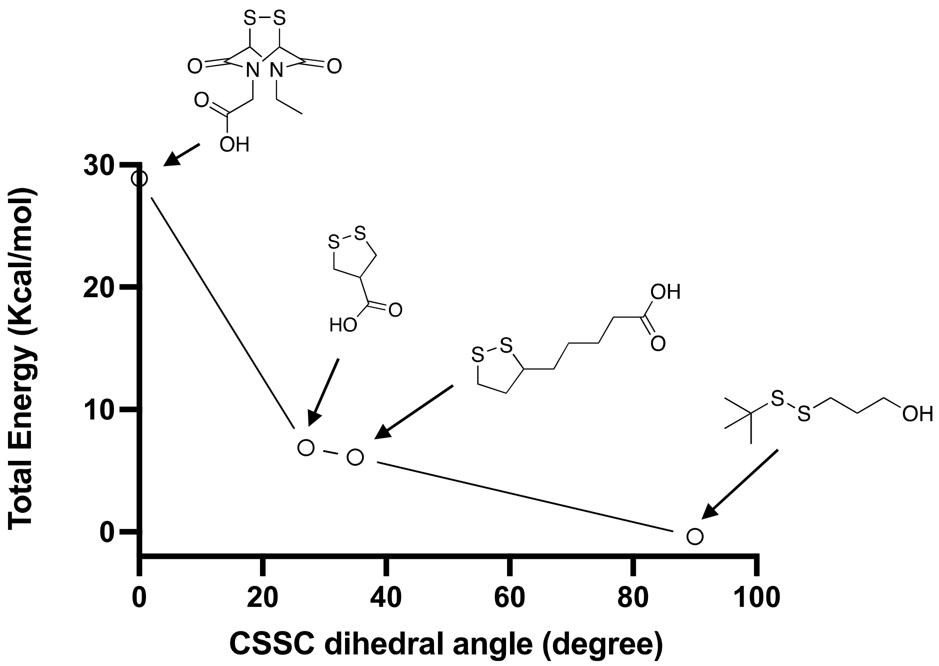

Figure S18. Energy profile for different disulfide unit.
